# Breaking barriers: pCF10 type 4 secretion system relies on a self-regulating muramidase to modulate the cell wall

**DOI:** 10.1128/mbio.00488-24

**Published:** 2024-06-28

**Authors:** Wei-Sheng Sun, Gabriel Torrens, Josy ter Beek, Felipe Cava, Ronnie P.-A. Berntsson

**Affiliations:** 1 Department of Medical Biochemistry and Biophysics, Umeå University, Umeå, Sweden; 2 Wallenberg Centre for Molecular Medicine and Umeå Centre for Microbial Research, Umeå University, Umeå, Sweden; 3 Department of Molecular Biology and Laboratory for Molecular Infection Medicine Sweden, Umeå Centre for Microbial Research, SciLifeLab, Umeå University, Umeå, Sweden; Case Western Reserve University School of Medicine, Cleveland, Ohio, USA

**Keywords:** Type 4 Secretion System, cell wall, Gram-positive bacteria, pCF10, integrated structural biology

## Abstract

Conjugative type 4 secretion systems (T4SSs) are the main driver for the spread of antibiotic resistance genes and virulence factors in bacteria. To deliver the DNA substrate to recipient cells, it must cross the cell envelopes of both donor and recipient bacteria. In the T4SS from the enterococcal conjugative plasmid pCF10, PrgK is known to be the active cell wall degrading enzyme. It has three predicted extracellular hydrolase domains: metallo-peptidase (LytM), soluble lytic transglycosylase (SLT), and cysteine, histidine-dependent amidohydrolases/peptidases (CHAP). Here, we report the structure of the LytM domain and show that its active site is degenerate and lacks the active site metal. Furthermore, we show that only the predicted SLT domain is functional *in vitro* and that it unexpectedly has a muramidase instead of a lytic transglycosylase activity. While we did not observe any peptidoglycan hydrolytic activity for the LytM or CHAP domain, we found that these domains downregulated the SLT muramidase activity. The CHAP domain was also found to be involved in PrgK dimer formation. Furthermore, we show that PrgK interacts with PrgL, which likely targets PrgK to the rest of the T4SS. The presented data provides important information for understanding the function of Gram-positive T4SSs.

**IMPORTANCE:**

Antibiotic resistance is a large threat to human health and is getting more prevalent. One of the major contributors to the spread of antibiotic resistance among different bacteria is type 4 secretion systems (T4SS). However, mainly T4SSs from Gram-negative bacteria have been studied in detail. T4SSs from Gram-positive bacteria, which stand for more than half of all hospital-acquired infections, are much less understood. The significance of our research is in identifying the function and regulation of a cell wall hydrolase, a key component of the pCF10 T4SS from *Enterococcus faecalis*. This system is one of the best-studied Gram-positive T4SSs, and this added knowledge aids in our understanding of horizontal gene transfer in *E. faecalis* as well as other medically relevant Gram-positive bacteria.

## INTRODUCTION

Enterococci are Gram-positive (G^+^) bacteria commonly found in the environment and animal microbiomes. Enterococci are also opportunistic pathogens that are prevalent in hospital-acquired infections (1–3). These bacteria efficiently form biofilms (4, 5), and are often resistant to multiple antibiotics, including last-resort antibiotics such as vancomycin and linezolid (6, 7). They are also highly adept in transferring their resistance to other bacteria via horizontal gene transfer, usually via conjugation. Conjugation is mediated by type 4 secretion systems (T4SSs) that transfer DNA and protein(s) from a bacterial donor to a recipient cell. T4SSs are multi-protein complexes that span across the bacterial cell membrane(s) and cell wall. During the past years, our knowledge of T4SSs has greatly expanded, with published structures of the Dot/Icm system from *Legionella pneumophila*, the Cag system from *Helicobacter pylori,* and the T4SSs from the pKM101, R388, and F plasmids (all from *Escherichia coli*) (8–12). For a recent review of G^−^ T4SSs and their structure and function, please see Costa et al. (13). The best-known G^+^ T4SSs are encoded on the following plasmids: pCF10 and pIP501 from *Enterococcus faecalis*, and pCW3 from *Clostridium perfringens* (14–16). Among these, the T4SS from the single, tightly regulated, operon on pCF10 is likely the best characterized (14, 17–19). We and others have previously characterized the adhesin proteins that facilitate mating pair formation (20–22), as well as the DNA transfer and replication proteins (23–25). However, compared to the information from G^−^ T4SS systems, we still have only a very limited insight into G^+^ T4SSs (15).

One of the major differences between G^−^ and G^+^ bacteria is the thickness of the peptidoglycan (PG) layer that makes up the cell wall. While G^−^ cell walls are usually only a few nanometers thick (up to 4 nm in *E. coli*) (26), the cell walls of G^+^ bacteria are 30–100 nm (27). The cell wall of *E. faecalis* has been reported to be ca. 40 nm thick (28). In all cases, this cell wall forms a barrier that must be crossed during conjugation. In the characterized G^−^ systems the T4SSs span over the inner membrane, the periplasmic space (including the PG), and the outer membrane. In G^+^ systems this is predicted to be very different, with the T4SS channel spanning the single membrane but not through the entire PG layer. It is, therefore, not surprising that the PG-degrading enzymes that create specific lesions in the cell wall differ between G^−^ and G^+^ T4SSs. In G^−^ systems, the PG-degrading enzymes are usually soluble proteins that are secreted to the periplasm and needed to create lesions in the PG to allow for the T4SS channel assembly (29–31). In G^+^ systems, these enzymes are usually anchored to the cell membrane via a transmembrane helix and are thought to be required to create defined lesions in the PG layer to allow substrate transfer (32, 33). In T4SSs, these hydrolases are divided into four classes, ALPHA, BETA, and DELTA, which are found in G^+^ T4SSs, and the GAMMA class, which is present in G^−^ T4SSs (34). The pCF10-encoded PrgK is essential for conjugation and belongs to the DELTA family of larger PG-degrading enzymes (34). Previous work has indicated that PrgK consists of an intracellular N-terminal domain, a transmembrane helix, and an extracellular part consisting of a metallo-peptidase (LytM), a soluble lytic transglycosylase (SLT), and a cysteine, histidine-dependent amidohydrolases/peptidases (CHAP) domain (32). While all three extracellular domains can bind PG *in vitro* and their ectopic over-expression led to cell-morphology defects in *E. coli*, PG hydrolysis activity could only be measured for the SLT domain (32). However, PrgK variants lacking the SLT domain were reported to partially complement a *prgK* knockout strain *in vivo*, indicating that at least one other domain retains hydrolysis activity. It has been unclear how PrgK is regulated or how it interacts with the remaining T4SS channel proteins.

Here, we report the crystal structure of the LytM domain and combine AlphaFold models with biochemistry to analyze the remaining part of the extracellular domains. AlphaFold modeling suggested that the extracellular domain of PrgK interacts with that of PrgL, another pCF10-encoded T4SS protein that plays a role in T4SS biogenesis (35). We confirm this interaction *in vitro* and show that PrgK can also dimerize in a redox-dependent manner. Finally, we characterized PrgK activity *in vitro* and revealed that the predicted SLT domain exhibits muramidase instead of the previously predicted lytic transglycosylase activity and that this domain is negatively regulated by the other two extracellular domains: LytM and CHAP.

## RESULTS

### X-ray structure and AlphaFold2 modeling

**Fig 1:**
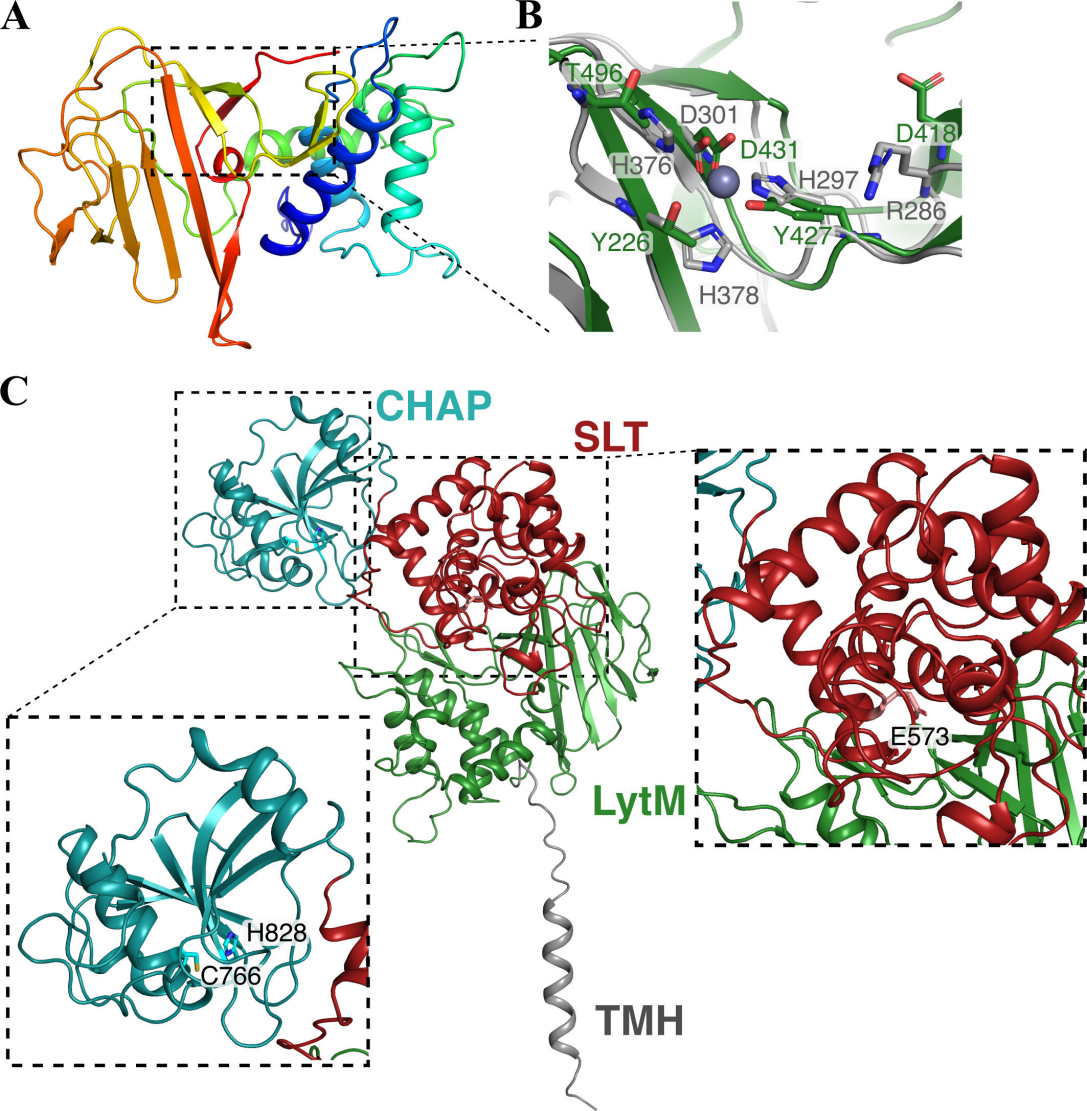
Structure of PrgK. (**A**) Cartoon representation of the PrgK_LytM_, colored from N-terminal (blue) to C-terminal (red). The active site is indicated by the dashed box and further enlarged in panel B. (**B**) Comparison of the active site of PrgK_LytM_ (green) and ShyA from *Vibrio cholerae* (gray). Key residues in the active site of ShyA, important for either coordination of the Zn^2+^ (gray sphere) or other activity are shown as sticks, together with the corresponding residues from PrgK_LytM_. (**C**) AlphaFold2 model of PrgK, showing the transmembrane helix and the three extracellular domains. The intracellular domain is less well predicted and is therefore not shown for clarity. The active sites of the CHAP and SLT domains, with their respective conserved residues, are further highlighted in the zoomed-in boxes.

To unravel the function and regulation of PrgK as the cell wall remodeling enzyme, we attempted to overexpress and purify full-length PrgK from *E. coli*. Unfortunately, this only gave extremely low yields. Therefore, we directed our efforts toward the extracytoplasmic region (PrgK_EC_), as well as its individual LytM, SLT, and CHAP domains (32). All these components were successfully produced and purified to homogeneity (Fig. S1). Crystallization trials yielded crystals of the PrgK_LytM_ domain, which were used to solve the LytM structure to 1.5 Å (Fig. 1A; Table 1). In agreement with bioinformatic predictions (32), our crystal structure confirms that the active site of LytM is degenerate as it lacks the conserved HXXXD and HXH motifs required for zinc ion coordination at the catalytic site (36, 37) (Fig. 1B). This is not the only characterized LytM domain with a degenerate active site. One of the top hits from DALI (38) and Foldseek (39) searches with PrgK_LytM_ is the regulatory protein DipM with an r.m.s.d. of 2.68 Å (PDB code: 7QRL) from *Caulobacter vibrioides*, which is an activator of the cell wall remodeling amidase AmiC and the lytic transglycosylase SdpA (40–42) (Fig. S2A). Sequence alignment of PrgK_LytM_ homologs shows very low sequence identity, with only the region encompassing the HXXXD and HXH motifs being conserved. The His and Asp residues that are important for catalysis are only conserved in active homologs, but not in PrgK_LytM_ and other degenerate homologs (Fig. S2B). In conclusion, the LytM domain of PrgK has a degenerate active site and might play a regulatory role.

**TABLE 1:** Data collection and refinement statistics

Data collection summary	PrgK_LytM_
Resolution range	49.13–1.5 (1.554–1.5)
Space group	P1
Cell dimensions	
*a*, *b*, *c* (Å)	53.32, 53.69, 56.35
α, β, γ (°)	63.3, 70.4, 70.2
Total reflections	144,756 (14,115)
Unique reflections	76,670 (7,437)
Multiplicity	1.9 (1.9)
Completeness (%)	93.35 (90.66)
Mean I/sigma (I)	6.98 (2.9)
R-meas	0.12 (0.42)
CC(1/2)^*a*^	0.977 (0.942)
Refinement summary	
*R*_work_	0.177 (0.259)
*R*_free_	0.208 (0.289)
Number of non-hydrogen atoms	4,961
Protein	4,268
Solvent	693
RMS (bonds)	0.012
RMS (angles)	1.09
Ramachandran favored (%)	97.45
Ramachandran allowed (%)	2.55
Ramachandran outliers (%)	0.00
Average B-factor	24.94
Macromolecules	23.30
Solvent	35.02

^*a*^ Statistics for the highest-resolution shell are shown in parentheses.

To obtain a structural model for full-length PrgK, we used AlphaFold2 (AF) (43). The AF model exhibited low certainty for the intracellular domain but did predict an alpha helix for the transmembrane helix as predicted by TOPCONS (44) (residues 254–274) (Fig. 1C). AF confidently predicted the structures of the extracytoplasmic domains, PrgK_EC_, including the relative positioning of the LytM and SLT domains (Fig. 1C; Fig. S3A and B). However, investigating the model shows that only very few interactions are present between LytM and the SLT domain, indicating that the spatial relationship between the extracellular domains is very uncertain. Conserved residues in the active sites of the SLT and CHAP domains are indicated in Fig. 1C. The crystal structure of LytM superimposes well onto the AF model with an r.m.s.d. of 0.43 Å.

### PrgK interacts with PrgL *in vitro*

**Fig 2:**
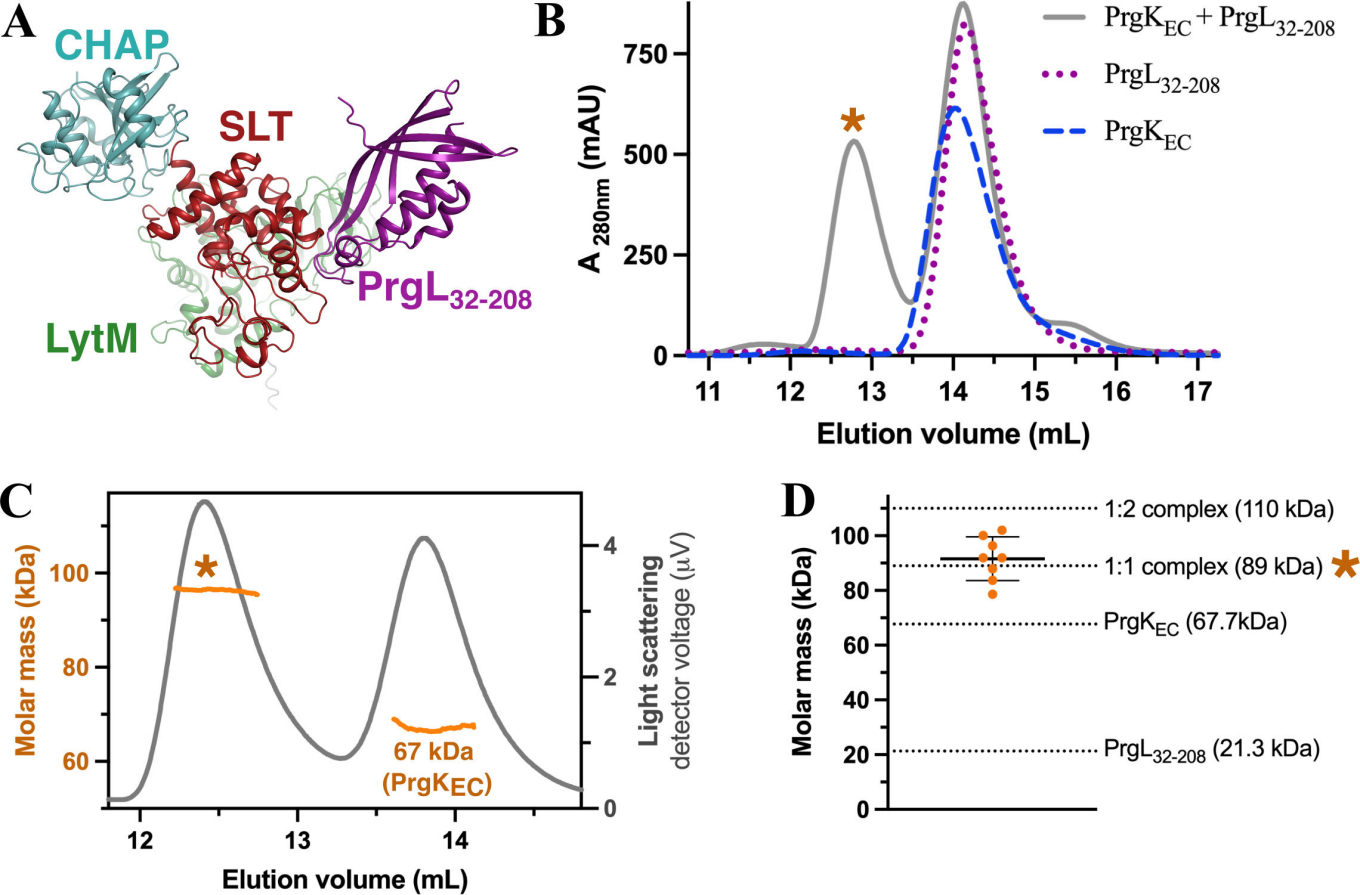
PrgK interacts with PrgL. (**A**) AlphaFold2 model of the PrgK:PrgL heterodimer predicts that PrgL_32-208_ binds to the SLT domain of PrgK. (**B**) Elution profile from size exclusion chromatography of PrgK_EC_, PrgL_32-208_, or a (1:1) mixture of both. The elution profile of the mixture shows an additional peak at an elution volume of 12.7 mL, indicated by an orange star. (**C**) Representative size-exclusion chromatography connected to multi-angle light scattering (SEC-MALS) analysis of the PrgK_EC_ + PrgL_32-208_ (1:1) mixture, with the determined molecular mass, indicated as orange lines for both the earlier eluting complex and non-complex (corresponding to the molecular mass of PrgK_EC_ 67.7 kDa) peaks. (**D**) Calculated molecular mass of the eluting complex from all eight SEC-MALS runs. Although there was some variation in the determined mass, the average was 91.5 ± 8 kDa (average and standard deviation of 8 independent measurements), which corresponds well to a 1:1 complex of the two components (PrgK_EC_ is 67.7 kDa and PrgL_32-208_ is 21.3 kDa).

Next, we used AF to predict potential interacting partners for PrgK among the other T4SS proteins of pCF10 (17, 19). This analysis suggested a PrgK:PrgL heterodimer with reasonable confidence. In this AF model, multiple hydrogen bonds are formed between the SLT domain of PrgK and a loop region of PrgL (Fig. 2A; Fig. S4A and B). The analogous T4SS proteins from the conjugative plasmid pIP501, TraG, and TraM, have previously been reported to interact (45), further strengthening this predicted interaction.

To validate the predicted interaction between PrgK and PrgL, we tested whether the purified extracellular domains of the two proteins (PrgK_EC_ and PrgL_32-208_) could form a complex and co-elute from size exclusion chromatography (SEC). PrgL_32-208_ forms elongated dimers in solution (35), which gives it a similar elution volume as PrgK_EC_ (Fig. 2B, ~14 mL elution volume). However, the elution profiles of PrgK_EC_ and PrgL_32-208_ mixtures show an additional peak that elutes earlier and that contains both proteins, thus confirming the predicted interaction (Fig. 2B; Fig. S4C, ~12.5 mL elution volume). To define the stoichiometry of this complex of PrgK_EC_ and PrgL_32-208_, we performed size-exclusion chromatography connected to multi-angle light scattering (SEC-MALS). The determined molecular mass of the complex eluting around 12.5 mL was 91.5 ± 8 kDa (average and standard deviation of 8 independent measurements), which corresponds to a 1:1 complex of the two components (Fig. 2C and D, PrgK_EC_ is 67.7 kDa and PrgL_32-208_ is 21.3 kDa). This shows that binding to PrgK disrupts the dimeric state of PrgL_32-208_ (35). Since the AF model suggested that PrgL_32-208_ interacted primarily with the SLT domain, we also tested whether a mixture of SLT + PrgL_32-208_ co-eluted on SEC. However, no complex was observed in those experiments (Fig. S4D), indicating that PrgL_32-208_ interacts with more than just the SLT domain of PrgK_EC_.

### Dimerization of PrgK *in vitro* is dependent on Cys766 in the CHAP domain

**Fig 3:**
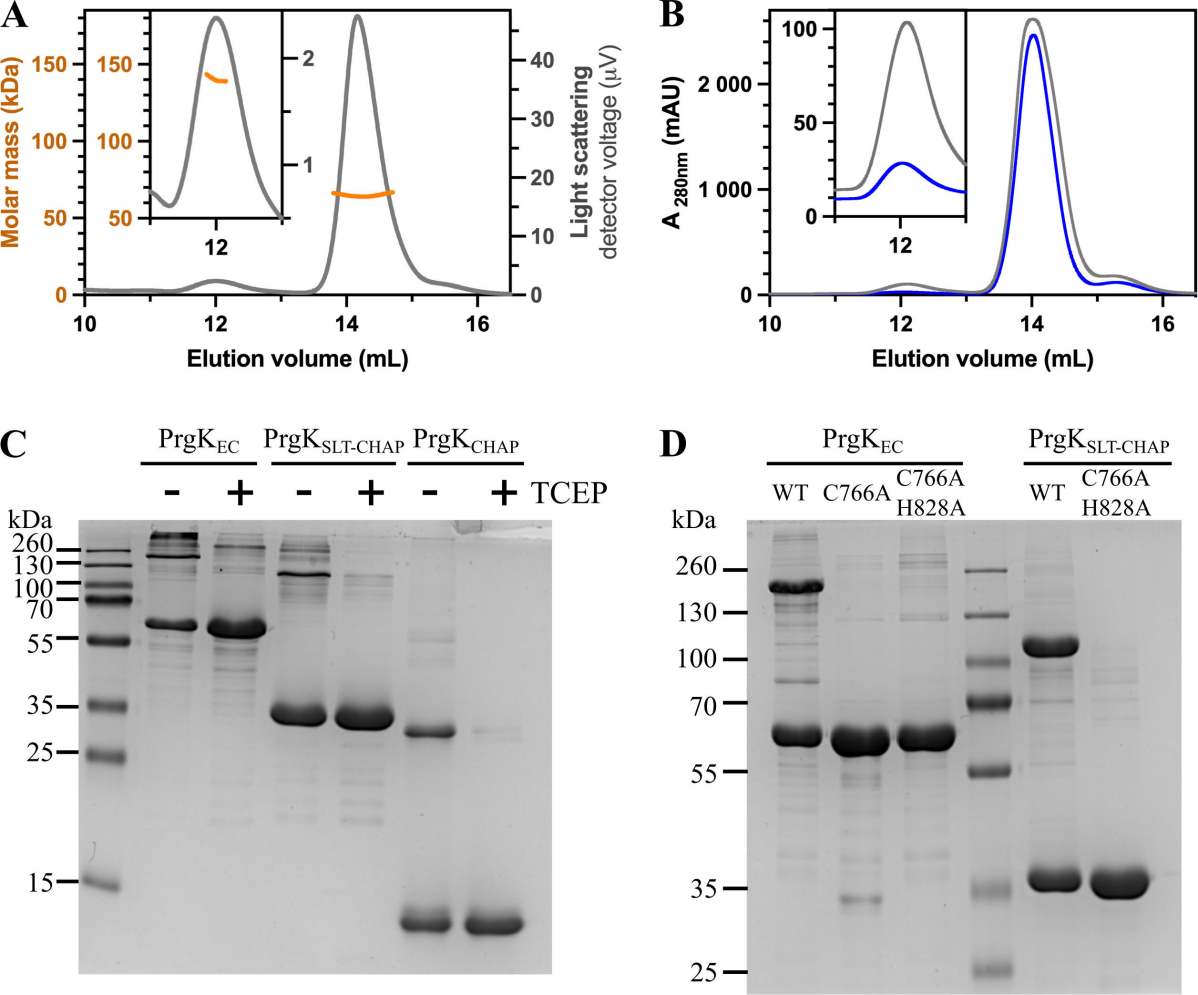
Dimerization of PrgK. (**A**) Representative SEC-MALS analysis of PrgK_EC_ (with a theoretical monomeric weight of 67.7 kDa), with the determined molecular mass, indicated as orange lines for both the dimeric (shown enlarged in the inset) and monomeric peaks. (**B**) SEC analysis of PrgK_EC_ wild type (gray line) and PrgK_EC:C766A_ (blue line) shows that the dimeric peak (shown enlarged in the inset) is substantially reduced in the C766A variant. (**C**) Non-reducing SDS-PAGE of purified PrgK samples with or without the addition of the reducing agent tris(2-carboxyethyl)phosphine (TCEP). Bands running at roughly the expected height of a dimer are seen for PrgK_EC_ (68 kDa), PrgK_SLT-CHAP_ (37 kDa), and PrgK_CHAP_ (16 kDa), and in all cases, these are significantly decreased upon the addition of TCEP. (**D**) Modifying cysteine 766 in the CHAP domain to an alanine prevents the formation of the dimer band in non-reducing SDS-PAGE.

During the purifications of PrgK_EC_, we consistently observed a less intense peak that eluted earlier from the SEC. Since the corresponding fraction only contained PrgK_EC_ (Fig. S1A), we speculated that this could correspond to a PrgK_EC_ dimer. This was verified by SEC-MALS experiments, which determined the molecular mass of the two eluting peaks to 64 ± 1 kDa and 144 ± 4 kDa (average and standard deviation of 3 independent measurements) (Fig. 3A). This corresponds well to the predicted molecular weight of the PrgK_EC_ monomer (67.7 kDa) and dimer (135 kDa).

We then investigated whether the partial dimerization was mediated by a disulfide bond. There is only a single cysteine in PrgK_EC_, which is found in the predicted active site of the CHAP domain (C766). Therefore, we created and purified a PrgK_EC_ variant where C766 was replaced by an alanine. PrgK_EC_ and PrgK_EC:C766A_ were loaded at similar concentrations (8.6 mg/mL) on SEC to compare their elution profiles. This showed a large reduction in the dimeric peak for the C766A variant (Fig. 3B). To further verify the role of this disulfide bond, we ran immobilized metal affinity chromatography (IMAC) purified protein fractions on a non-reducing SDS-PAGE. Without the addition of reducing agents, we indeed observe both a monomeric and a dimeric band of PrgK_EC_. Importantly, the dimeric band disappears upon the addition of the reducing agent TCEP to the loaded sample (Fig. 3C). PrgK_SLT-CHAP_ (37.4 kDa) and PrgK_CHAP_ (16.3 kDa) were also tested, as C766 is present in the CHAP domain, and showed the same redox-dependent oligomerization (Fig. 3C). The dimeric band on non-reducing SDS-PAGE could not be observed for the C766A variants (Fig. 3D). Taken together this data indicates that C766 can form disulfide bonds and aid in PrgK dimerization. The addition of PrgL_32-208_ did not alter the dimerization properties of PrgK_EC_ in any way (Fig. S4E).

### The SLT domain of PrgK has muramidase activity and it is regulated by the other two EC domains

**Fig 4:**
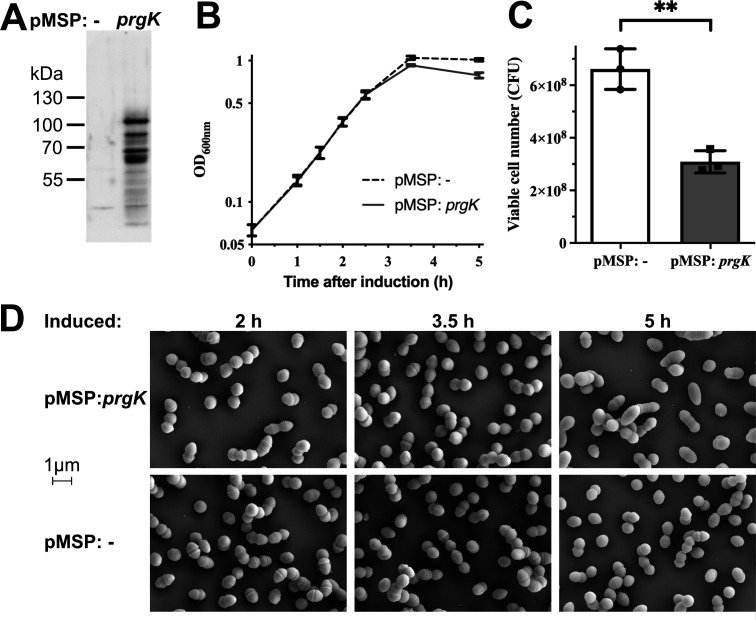
Ectopic expression of PrgK in the absence of other T4SS components. Comparison of *E. faecalis* OG1RF strains harboring a PrgK-expressing plasmid or an empty vector (−). (**A**) PrgK production analyzed by Western blot. (**B**) Growth curves of the two strains. Expression was induced at the beginning of incubation (0 h). (**C**) The viable cell numbers are 3.5 h after induction. Statistic significance was analyzed by an unpaired *t*-test analysis and gave a *P* value of 0.002. (B + C) Data from three independent experiments and error bars indicate the standard error of the mean. (**D**) Representative images of both strains under scanning electronic microscopy (scanning EM) at 30,000× magnification 2, 3.5, or 5 h after induction.

Periplasmic expression of the extracellular part of PrgK or its domains has been described to impair cell growth and lead to cell defects in *E. coli* due to extensive peptidoglycan degradation (32). However, there are often mechanisms in place to prevent autolytic enzymes from degrading the cell wall in their native host (46, 47). We therefore wanted to investigate the impact of PrgK expression on *E. faecalis* cells. Full-length PrgK was ectopically overexpressed in the OG1RF strain of *E. faecalis* in the absence of other T4SS proteins (Fig. 4A). In contrast to the findings in *E. coli*, no major effects were found upon overexpression of PrgK in *E. faecalis*. There were small effects on cell growth and cell viability (Fig. 4B and C), but only minor phenotypic differences could be observed by scanning electron microscopy. After 5 h we did observe evidence of lysed cells and a small subset of elongated cells as compared to the control cells, but no differences were observed at earlier time points (Fig. 4D). We conclude that PrgK overexpression has a small, but significant, impact on *E. faecalis* viability, but does not cause any drastic effect on the morphology of these cells.

**Fig 5:**
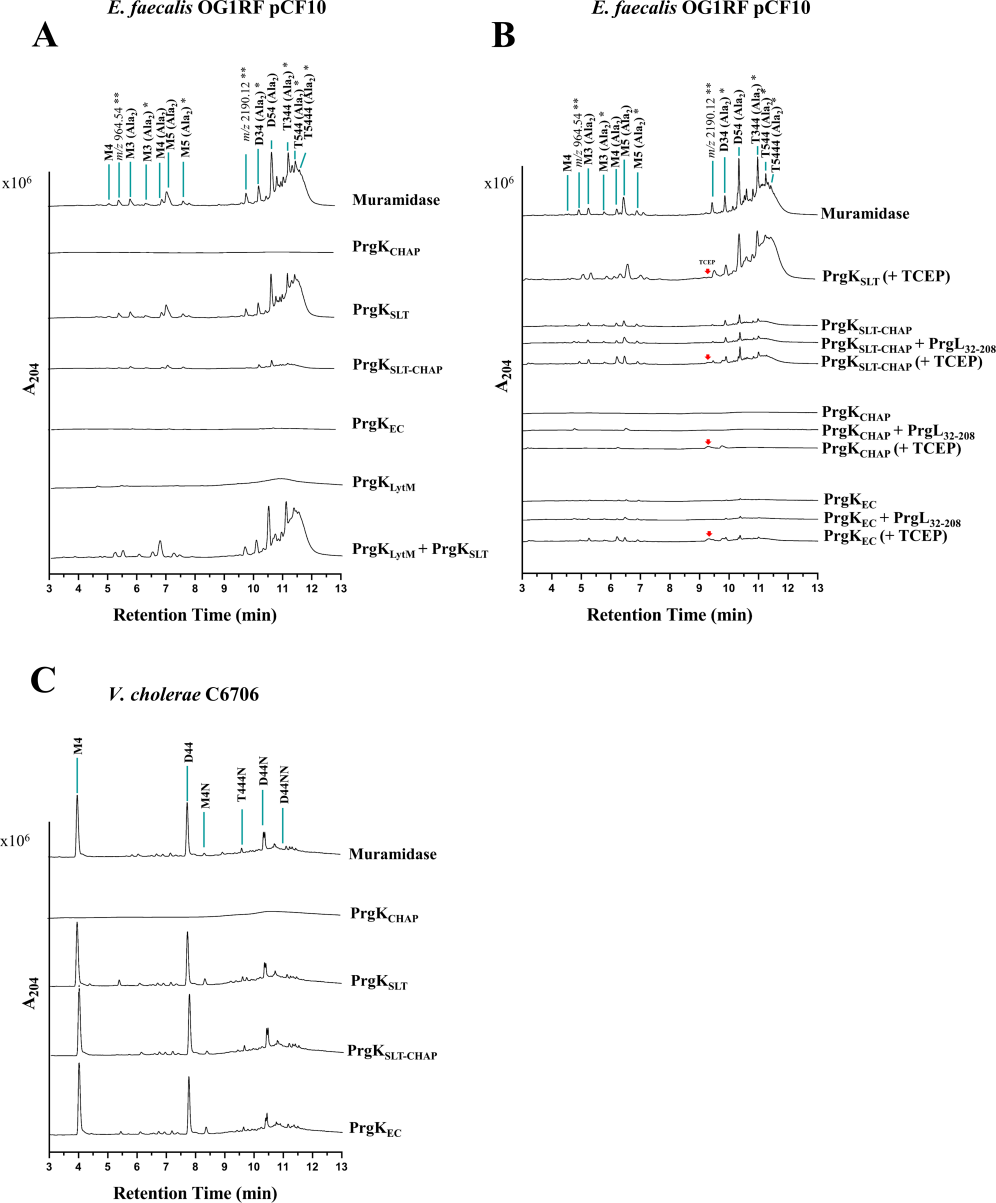
Comparing cell wall muropeptides generated by PrgK domains. (**A**) Chromatograms of muropeptides released following *E. faecalis* OG1RF:pCF10 PG treatment with the indicated PrgK enzyme variants. (**B**) Chromatograms of muropeptides released following *E. faecalis* OG1RF:pCF10 PG treatment with PrgL_32-208_ or TCEP. (**C**) *V. cholerae* C6706 PG treatment with the indicated PrgK enzyme variants. (**A–C**) Identified muropeptides are indicated above their corresponding peak. Unknown muropeptides are marked for their corresponding *m/z* values. Red arrows indicate the peak for TCEP. *, Gln/Glu. It is arbitrary to assign the amide and hydroxyl roles to either peptide stem. **, Precise structure unknown.

To characterize the enzymatic and regulatory properties of the PrgK extracellular domains (PrgK_EC_, PrgK_LytM_, PrgK_SLT_, PrgK_CHAP_, PrgK_SLT-CHAP_) we assayed their ability to digest the cell wall of *E. faecalis*. Experiments gave similar results when done with sacculi isolated from cCF10-induced *E. faecalis* OG1RF pCF10 as with the OG1RF pCF10:Δ*prgK* strain induced likewise (compare Fig. 5A and B with Fig. S5). This indicates that the activity of endogenous PrgK does not significantly alter the cell wall composition after pCF10 is induced by the pheromone cCF10 to initiate T4SS production. Consistent with a previous report (32), the SLT domain displayed PG-degrading activity as it digested *E. faecalis* PG into soluble muropeptides (PG fragments). However, in stark contrast with its prediction as a lytic transglycosylase, our ultra-performance liquid chromatography-mass spectrometry (UPLC-MS) analyses revealed that the PG fragments generated by PrgK_SLT_ were identical to that produced by muramidase (Fig. 5A; Fig. S5A). These fragments differ substantially from those generated by a standard SLT protein like the SLT from *E. coli* (Slt_70_), which is not active against *E. faecalis* PG (Fig. S6A and B).

Curiously, the activity of PrgK_SLT-CHAP_ was reduced compared to PrgK_SLT_, and PrgK_EC_ showed an even further reduction (Fig. 5A). To investigate if this reduction in PG degradation was due to the CHAP domain further degrading the products of the SLT domain, we constructed and purified variants of PrgK_SLT-CHAP_ and PrgK_EC_ in which the catalytic Cys766 and His828 residues of the CHAP domain were replaced by alanines. However, the muropeptide profiles of the PrgK_SLT-CHAP:C766A, H828A_ and PrgK_EC:C766A, H828A_ fractions after incubation with cell-wall extract are similar to their wild-type counterparts (Fig. S6A). In line with this result, the LytM and CHAP domains showed no activity against the sacculi (Fig. 5A), as reported earlier (32). Mixing individual LytM and SLT domains also did not give a difference in SLT activity, indicating that LytM PG binding does not act on SLT products by sequestration (Fig. 5A). These experiments therefore exclude the possibility of the hydrolytic activity of CHAP or LytM contributing to the diminished muropeptide profiles and instead suggest that CHAP and LytM strongly reduce the activity of the SLT domain in cell wall extracts from *E. faecalis*.

Since we found that PrgK can dimerize in a redox-dependent manner and that it can interact with PrgL, we also performed the activity measurements with PrgL_32-208_ present or under reducing conditions. According to the data analysis, the hydrolytic activities of PrgK_SLT-CHAP_ and PrgK_EC_ were not altered by PrgL_32-208_. PrgL_32-208_ also did not activate PrgK_CHAP_ (Fig. 5B). Addition of the reducing reagent TCEP, to remove any PrgK dimers, showed a small but significant increase in muramidase activity (twofold increase over no addition, Fig. 5B). No increase was observed when adding TCEP to PrgK_SLT_. This indicates that PrgK dimer formation, via the CHAP domain, plays at least a small role in regulating PrgK activity.

Since the effect of PrgK expression in *E. faecalis* cells (Fig. 4) differed substantially from previous reports with expression of PrgK domains in *E. coli* (32), we further wanted to investigate whether the activity of PrgK could be different on cell-wall substrates from G^+^ and G^−^ bacteria. Therefore, we also assayed the ability of PrgK_SLT_, PrgK_CHAP_, PrgK_SLT-CHAP_, and PrgK_EC_ to cleave sacculi from *Vibrio cholerae*, a G^−^ bacteria. This analysis further validated the muramidase-like activity of PrgK_SLT_. PrgK_CHAP_ did also not show any activity under the tested conditions with this substrate. However, much to our surprise, the activity of PrgK_SLT-CHAP_ and PrgK_EC_ were the same as PrgK_SLT_, indicating that the downregulation of the activity of the SLT-domain does not occur with Gram-negative cell wall substrates (Fig. 5C).

## DISCUSSION

**Fig 6:**
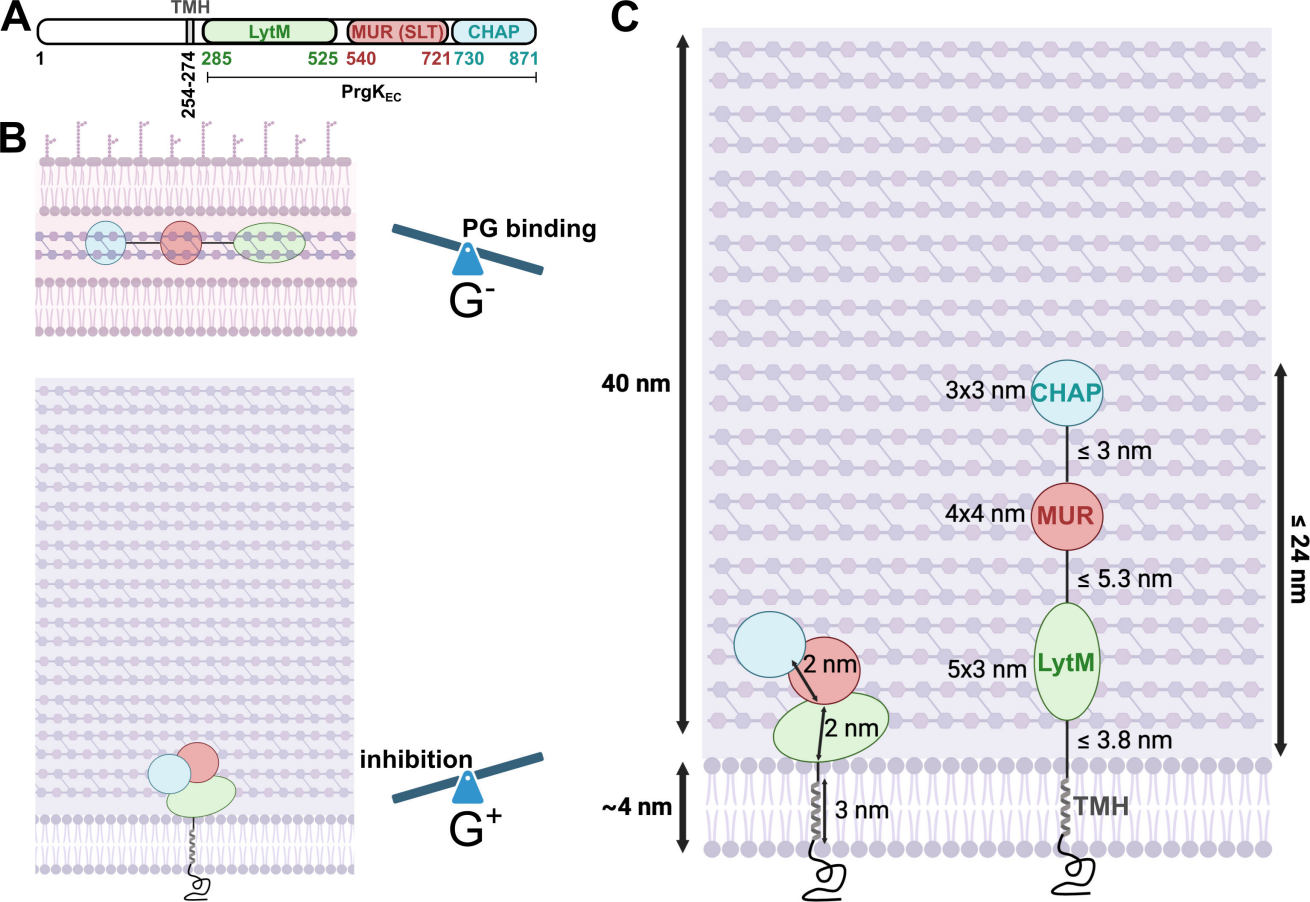
Schematic overview of PrgK and hypothesis of its differential regulation. (**A**) Domain organization of PrgK. The formally called SLT domain is renamed MUR domain, because of its muramidase activity. Domain boundaries were predicted by AlphaFold2 and differ slightly from previous predictions. (**B**) Model of PrgK_EC_ PG binding and hydrolysis in G^−^ or G^+^ bacteria. The LytM and CHAP domains can either bind to their PG substrates or interact with the MUR domain (formally termed SLT) thereby blocking its enzyme activity. In G^+^ bacteria, the MUR domain is inhibited by the CHAP and LytM domains as seen in *E. faecalis* cells and cell-wall extracts. In G^−^ bacteria, such as *V. cholerae* or *E. coli*, LytM and CHAP bind stronger to their PG substrate and do not inhibit the MUR domain. (**C**) Maximally extended PrgK (without unfolding the domains), can reach roughly the middle of the *E. faecalis* cell wall.

We have here studied the structure, function, and regulation of PrgK, the cell wall hydrolase of the T4SS from pCF10. Mass spectrometry analysis of cell wall extracts that were enzymatically cleaved by PrgK variants showed unexpectedly that the predicted SLT domain, named after its homology with soluble lytic transglycosylase enzymes, had instead a muramidase activity. Lytic transglycosylases cleave the 1,4-beta-linkages between the MurNAc and GlcNAc sugars with concomitant 1,6-cyclization of the MurNAc, resulting in glycan chains that terminate in a 1,6-anhydromuropeptide at the reducing end (48–52). Muramidases cleave the same bond in the PG but do so via a hydrolytic cleavage mechanism using completely different chemistry thereby producing MurNAc termini without the ring (53). We therefore rename the SLT domain in PrgK to the MUR (muramidase) domain (Fig. 6A). So far, all other T4SS PG-degrading enzymes have been reported to use proteins with lytic transglycosylase activity (34). However, to our knowledge, PrgK is the first T4SS PG-degrading enzyme from the DELTA family that has been characterized in detail.

When we understood that PrgK had muramidase activity, we revisited the AF model of the MUR domain and searched for homologs. Only one structural homolog was found in the PDB by using Foldseek, which is the geneproduct 13 (gp13) cell-wall degrading enzyme of bacteriophage Φ29 (54). gp13 consists of two domains, one N-terminal lysozyme-like domain that is homologous to the MUR domain (RMSD of 1.5 Å), and a C-terminal LytM domain (RMSD to PrgK_LytM_ of 2.4 Å) (Fig. S7A). The MUR domain of PrgK retains the same active site, including the catalytically important glutamic acid residue (Glu573), but compared to gp13 the MUR domain has extended loop regions that frame the active site rather differently (Fig. S7B). It will be interesting to in the future fully delineate the active site and study its enzymatic activity in detail. When searching against the AlphaFold DataBase (AFDB) (55) virtually all top hits are phage tail lysozyme domain-containing proteins. It is difficult to determine the evolution of proteins encoded on conjugative plasmids, but it is tempting to speculate that PrgK has picked up the MUR domain (and possibly also the LytM domain) from bacteriophages at some point during its evolution.

Previous work showed that *prgK_lytM_* could not complement a Δ*prgK* strain and the LytM domain was suggested to have a degenerate active site. Our crystal structure confirms this (Fig. 1). The *in vitro* analysis of the LytM domain, as well as the CHAP domain, also showed virtually no activity. This was surprising given that a PrgK variant lacking the MUR domain, but still having the LytM and CHAP domains could partially rescue a Δ*prgK* strain *in vivo* (32). Possibly, PrgK_CHAP_ has a low background level of activity *in vivo* that is sufficient to support conjugation. An analog of PrgK is TraG in the pIP501 T4SS, also from *E. faecalis*. TraG contains an SLT and CHAP domain but lacks a LytM (33). The SLT domain of TraG has been indicated to be a lytic transglycosylase since it is inhibited by specific LT inhibitors (33). Both TraG_SLT_ and TraG_CHAP_ domains have also been shown to have relatively limited activities against *E. faecalis* PG in the absence of mutanolysin, and have significantly increased digestion when they react together in *cis* (33). These properties of TraG are distinct from PrgK, which has three domains that do not react synergistically. This indicates substantial differences in how the two G^+^ T4SSs modify the cell wall in preparation for conjugation.

Our activity assays showed that PrgK_EC_, containing all three extracellular domains (LytM, MUR, and CHAP), only had a very low activity on *E. faecalis* cell-wall extracts (Fig. 5A). The presence of the other two domains thus downregulated the activity of the MUR domain, presumably by sterically blocking the active site. This is analogous to how some other cell-wall degrading enzymes are regulated, like Auto from *Listeria monocytogenes* or ShyA from *V. cholerae* (46, 47). The predicted AlphaFold2 model of PrgK_EC_ does not indicate that the active site of the MUR domain is blocked by the other domains. However, the PG substrates are large and there is quite some flexibility in the linker regions between the LytM, MUR, and CHAP domains that could allow for a reorganization of the three domains and a steric inactivation of the MUR domain. This hypothesis that CHAP and LytM downregulate the activity of the MUR domain is further supported by our phenotype analysis of an *E. faecalis prgK* overexpression strain (Fig. 4). Our experiments could only detect minor effects on cell growth and cell shape upon *prgK* overexpression. If the MUR domain in PrgK had been fully active in these experiments, we would likely have seen much larger changes as previously reported for *E. coli* expression (32). Interestingly, this downregulation of the MUR domain by the other two domains is not seen when a cell wall extract from *V. cholerae*, a G^−^ bacteria, is used. There, the MUR domain has the same activity in isolation as in PrgK_EC_. From previous work, we know that both the LytM and CHAP domains have the potential to bind peptidoglycan (PG) (32). We, therefore, hypothesize that the LytM and CHAP domains are in an equilibrium between interacting with the MUR domain, thereby sterically blocking its active site, and interacting with the PG (Fig. 6B). While the muramidase we use as control is highly promiscuous (56), previous data has shown that muramidases are often inhibited by O-acetylated sugars, such as those found in *E. faecalis* PG (57). This inhibition, or lack of binding, would not be observed in *V. cholerae* PG which lacks such O-acetylation. A similar effect could potentially play a role with the LytM and CHAP domains, leading to a shift in the equilibrium toward more PG interaction in *V. cholerea*. This would free the active site of the MUR and allow it to bind and digest the PG of these G^−^ bacteria (Fig. 6B). The regulation of the MUR domain of PrgK, by the CHAP and LytM domains, that is observed with *E. faecalis* cell-wall extract*,* is likely important to prevent extra toxicity to the donor cells upon induction of the T4SS. It also seems tuned to the host species, as the regulation is lost on the cell wall from G^−^ species. Presumably, PrgK gets activated by another component at an early point after T4SS induction, but it’s not yet clear how this occurs. These aspects will be interesting to define in future work.

Analogous to the interaction of TraG and TraM in pIP501 T4SS (45), AlphaFold predicted the interaction of PrgK with PrgL, which we could confirm *in vitro* using size-exclusion chromatography (Fig. 2). However, PrgL did not alter the activity of PrgK (Fig. 5). We speculate that PrgL binds PrgK to localize it to the T4SS channel during channel assembly. This is in line with the previous hypothesis that PrgL, as other VirB8-like proteins, acts as a scaffold during the biogenesis of the T4SS (35). The AlphaFold model predicting the interaction between PrgK and PrgL suggests that PrgL binds to the MUR domain. While we could show that PrgL binds strong enough to PrgK_EC_ to co-elute on SEC, this was not observed for PrgL and the MUR domain (Fig. S4D). This suggests that the MUR domain alone is not sufficient to stabilize the interaction between PrgK and PrgL, and implies the involvement of other parts of PrgK_EC_. PrgL binds to PrgK in a 1:1 ratio, indicating that the dimer of PrgL is broken upon binding to PrgK. However, the AF model alone cannot explain why the PrgL dimer gets disrupted.

During the purification of PrgK_EC_, we noted that a small amount of the purified protein eluted earlier from SEC. This fraction was confirmed to be a redox-dependent dimer of PrgK_EC_, that was formed by disulfide bonds between the single cysteine, C766, from two CHAP domains (Fig. 3). C766 is an integral part of the active site of the CHAP domain and dimer formation via this residue will therefore likely shut down any potential activity that the CHAP domain has. Our cell wall activity data could not detect any activity for the CHAP domain. This absence of enzymatic activity makes sense if the main function of the CHAP domain is not hydrolysis, but instead regulation and dimer formation.

A large question in the field of G^+^ T4SS is how the substrate gets transported into the recipient cell. No pili exist in these systems, but instead, cell wall hydrolases have been suggested to form a discrete lesion in the cell walls of both cells, basically providing a physical tunnel of sorts between the mating pair. However, structural analysis of PrgK reveals that even if the protein were completely extended (without unfolding the individual domains), it would only reach roughly the middle of the cell wall of the *E. faecalis* donor cell (Fig. 6C). Thus, we deem it very unlikely that PrgK could create a defined substrate tunnel between the donor and recipient bacteria. Instead, we propose that the main function of PrgK is to create enough space in the cell wall of the donor cell to allow for channel assembly. This means that it remains unclear how the substrate transfers with a high efficiency from the donor cell, over two cell wall barriers and finally entering the recipient cell by somehow passing over its membrane. Answering this question will be of great interest in future research.

To conclude, we here show that PrgK, an essential cell wall degrading enzyme of the pCF10 T4SS, has muramidase activity instead of the lytic transglycosylase activity that has been shown for other T4SS cell wall degrading enzymes. Only the MUR domain has measurable activity *in vitro*, while the other two extracellular domains of PrgK instead have a regulatory function. This results in the autoregulation of PrgK hydrolysis activity, which likely is important to prevent toxicity for the donor cell. Finally, we have also shown that PrgK interacts with PrgL, which sheds further light on the assembly mechanisms of Gram-positive T4SSs.

## MATERIALS AND METHODS

### Strains and culture conditions

See Table S1 for detailed plasmid and strain information. In brief, *E. coli* Top10 was the host strain for plasmid construction and propagation, and *E. coli* BL21(DE3) was the host strain for isopropyl β-D-thiogalactopyranoside (IPTG)-inducible protein over-expression. *E. coli* strains were cultivated in Luria Bertani (LB) broth with necessary selection antibiotics at 37°C with agitation. *E. faecalis* strains were cultured with brain-heart infusion (BHI) for overnight incubation or in tryptic soy broth without dextrose (TSB-D) with antibiotics required for plasmid selection. Concentrations of antibiotics for *E. coli* selection were as follows: kanamycin (50 µg/mL) and chloramphenicol (25 µg/mL). In *E. faecalis* cultures, antibiotics were used in the following concentrations: tetracycline (10 µg/mL), fusidic acid (25 µg/mL), erythromycin (100 µg/mL), spectinomycin (1,000 µg/mL). Plasmid transformations to *E. coli* were carried out by standard heat shock, whereas *E. faecalis* strains were transformed by electroporation (58).

Overnight cultures of *E. faecalis* OG1RF transformed with empty pMSP3545S nisin-inducible vector or with the same vector harboring *prgK* (59), were inoculated in fresh TSB-D medium with selection antibiotic (erythromycin) and 50 ng/mL nisin to an initial OD_600_ of 0.05. After incubating at 37°C with agitation for the indicated time (1, 1.5, 2, 2.5, 3.5, and 5 h), optical density was measured again for the growth curve. For *E. faecalis* cell viability, cell cultures were harvested after 3.5 h. Individual samples were serially diluted and then plated out on BHI agar plates. The resulting plates were incubated at 37°C for colony-forming unit enumeration.

### Analysis of PrgK expression by western blot

*E. faecalis* cells cultured and induced as described in the last paragraph were harvested at 3.5 h post-induction by spinning at 13,000 × *g* for 5 min at 4°C. The cell pellets were then resuspended in lysozyme buffer (10 mM Tris, pH 8.0, 1 mM EDTA, 25% sucrose, 15 mg/mL lysozyme) and incubated for 30 min at 37°C. The lysozyme-treated bacterial cells were then mixed with protein sample buffer and boiled at 100°C for 12 min. Subsequently, the samples were run on a 10% SDS-PAGE. Proteins went through electrophoresis and were transferred to a nitrocellulose paper, which was probed with the PrgK antibody produced in rabbits. HRP-conjugated anti-rabbit antibody was applied to the membrane for signal augmentation and then developed with ECL reagents for image development.

### Scanning electron microscopy

*E. faecalis* cells for scanning EM were cultured and induced as described for the growth curve measurements above. After 2, 3.5, or 5 h incubation, 5 mL of the cultures were spun down at 3,000 *× g*, 4°C for 10 min. Pellets were resuspended in 400 µL 0.1 M Na-phosphate buffer (pH 7.4) and transferred to a 1.5 mL tube. The cells were again pelleted, but now at 20,000 *× g*, 4°C, 10 min. All supernatant was removed, and the pellets were carefully resuspended in 200 µL 0.1 M Na-phosphate buffer (pH 7.4) (by both pipetting and vortexing). Afterward, 200 µL 5% glutaraldehyde in 0.1 M Na-phosphate buffer (pH 7.4) was added, mixed by pipetting up and down to crosslink the cells, and incubated for several hours at 4°C. The mixtures were then further incubated overnight at 4°C on top of polylysin-coated coverslips that were placed at the bottom of a 24-well plate. Samples were washed 3 times with 0.1 M phosphate buffer and then dehydrated with graded series (50%, 70%, 80%, 90%, 100%) of ethanol. They were further critical point dried with Leica EM300. The samples were coated with a 5 nm layer of Pt (Quorom Q150T ES) and imaged using a field emission scanning electron microscope (FESEM; Carl Zeiss Merlin) at an accelerating voltage of 5 kV and probe current of 120 pA.

### Cloning and plasmids

To characterize the extracytoplasmic region of PrgK, DNA sequences covering different fragments of PrgK were amplified for protein expression and subsequent studies. Domain boundaries were identified using Phyre2 and RaptorX (60, 61). pMSP3545-prgK (32) was used as template along with the corresponding primer pairs (Table S1) to amplify PCR products covering sequences coding for PrgK_EC_ (residues 273-871), PrgK_SLT-CHAP_ (residues 530-871), PrgK_LytM_ (residues 273-529), PrgK_SLT_ (residues 539-723), and PrgK_CHAP_ (residues 723-871); and pMSP3545-prgK:_C766A H828A_ was the template for generating amplicons for PrgK_EC:C766A H828A_ and PrgK_SLT-CHAP:C766A H828A_. These PCR products were cloned into the pINIT_Kan vector for sequencing and then sub-cloned to p7X expression vectors (p7XC3GH and p7XNH3) following the guidelines of the FX-cloning system (62). p7XNH3-prgK_EC:C766A_ was constructed by the same site-directed mutagenesis protocol as previously described (20) with partial-overlapping primer pair PrgK_C766A_inv-F and PrgK_C766A_inv-R. Plasmids expressing the lytic transglycosylase *slt70* (from *E. coli*) and muramidase *XNR_0208* (from *Streptomyces albus*) were used from previous work (63, 64).

### Protein expression and purification

p7X expression plasmids harboring the His-tagged PrgK variants-encoding sequences were transformed to *E. coli* BL21(DE3). The transformants were firstly inoculated in LB broth for small-scale overnight incubation (37°C, 200 rpm), then diluted 1:100 in Terrific broth and further incubated at 37°C in the LEX bioreactor system (Epiphyte3). When the optical density reached 1.5, the incubation temperature was lowered to 18°C, and expression was induced by adding 0.1 mg/mL IPTG for 18 h. Afterward, cells were pelleted, resuspended in lysis buffer (50 mM Na_2_HPO_4_/NaH_2_PO_4_, 300 mM NaCl, 10 mM imidazole, pH 7.0) in a ratio of 7 g/100 mL and lysed at 4°C and 25 kPsi using a Cell Disruptor (Constant System). Cell debris was removed by centrifugation for 30 min at 13,000 × *g*, 4°C, and the supernatant was incubated at 4°C with 1.25 mL Ni-NTA agarose resin (Macherey-Nagel) for 1 h with agitation. By gravity, the Ni-NTA resin was washed with 40 column volumes of wash buffer (50 mM Na_2_HPO_4_/NaH_2_PO_4_, 300 mM NaCl, 50 mM imidazole, pH 7.0). The bound proteins were eluted from the column by incubation with PreScission protease in a 1:100 ratio for 20 h at 4°C, thereby removing the histidine affinity tag. Tag-free proteins were then loaded on a Superdex-200 Increase 10/300 Gl column (Cytiva) equilibrated in one of the following buffers. Buffer for PrgK_LytM_ was 50 mM HEPES, 500 mM NaCl, pH 7.5. For the individual fragments of PrgL_32-208,_ PrgK_EC_, PrgK_SLT-CHAP_, PrgK_SLT_, and PrgK_CHAP_ , a buffer of 100 mM citrate-citric acid, pH 5.0, 100 mM NaCl was used.

Slt70 and Muramidase were expressed and purified as previously described (63, 64). Briefly, expression was induced in LB cultures at the exponential phase with 1 mM IPTG for 2 h. Cells were harvested, resuspended in 150 mM Tris HCl pH 7.5, 150 mM NaCl, and stored at −20°C. After thawing on ice, cells were disrupted by passing through a French press twice. 6×His-tagged proteins were purified from cleared lysates (30 min, 100,000 *× g*) on nickel-nitrilotriacetic acid-agarose columns (QIAGEN), and eluted with 150 mM Tris-HCl (pH 7.5), 150 mM NaCl, 250 mM imidazole. The eluate was dialyzed for 12 h in 50 mM Tris-HCl (pH 7.5), 100 mM NaCl.

### Crystallization and structure determination

SEC-purified PrgK_LytM_ in 50 mM HEPES, 500 mM NaCl, pH 7.5, with a protein concentration of 15 mg/mL, was used for crystallization trials. Crystals were observed after 3–5 days, at 20°C by sitting drop vapor diffusion in a condition with 0.2 M ammonium sulfate, 0.1 M sodium acetate, pH 4.6, 30% (wt/vol) PEG 2000 MME, and a protein to reservoir ratio of 1:1 in the drop. Crystals were flash-cooled in liquid nitrogen. X-ray diffraction data were collected on beamline ID30B at the ESRF, France. The data were processed using XDS (65). The PrgK_LytM_ crystals belong to the space group of P1 and contain two molecules in the asymmetric unit. The crystallographic phase problem was solved by molecular replacement of PHASER (66), using the Alphafold2 model of LytM (residue 273-529) as a search model. Further model refinement was done by using PHENIX refine (67) and further modeled using COOT (68). The structure was refined to 1.5 Å with crystallographic *R*_work_/*R*_free_ values of 17.7%/20.8% (Table 1). The final PrgK_LytM_ model consists of residues 274-529 and was validated using MolProbity (69). Atomic coordinates and structure factors have been deposited in the Protein Data Bank (PDB Code: 8S0U).

### AlphaFold modeling

ColabFold (70) (v1.5.2) was used to generate predictions of full-length PrgK using default settings, whereas the prediction of PrgK-PrgL interaction was carried out with AlphaFold-Multimer using default settings.

### Size exclusion chromatography with multi-angle static light scattering

For analysis of the PrgK_EC_ dimer, 150–300 µL 9.6 mg/mL PrgK_EC_ (with a theoretical mass of 67.4 kDa) was loaded on a Superdex 200 Increase 10/300 Gl column, equilibrated in SEC buffer (100 mM citrate-citric acid, pH 5.0, 100 mM NaCl) via an ÄKTA Pure (Cytiva) that was coupled to a light scattering (Wyatt Treas II) and refractive index (Wyatt Optilab T-Rex) detector to determine the molecular weight of the two eluting components (at ~12 and ~14.2 mL) via multi-angle laser light scattering (SEC-MALS).

For analysis of the PrgK-PrgL complex, 3–4 mg/mL PrgK_EC_ was mixed with PrgL_32-208_ in a molar ratio of 1:1 or 1:3 and incubated for 15 min at 4°C. For three analyses, 200 μL of the 1:1 mixture was loaded directly on a Superdex 200 Increase 10/300 Gl column, equilibrated in SEC buffer (100 mM citrate-citric acid, pH 5.0, 100 mM NaCl) via an ÄKTA Pure (Cytiva) that was coupled to a light scattering (Wyatt Treas II) and refractive index (Wyatt Optilab T-Rex) detector to determine the molecular weight of the two eluting components (at ~12.5 and ~13.8 mL) via multi-angle laser light scattering (SEC-MALS). For a fourth analysis, the same was done, but then with the 1:3 mixture. For four additional analyses, a preparative SEC run on a similar column (Superdex 200 Increase 10/300 Gl) in the same SEC buffer with an Äkta Purifier (Cytiva) was done prior to the SEC-MALS analysis to isolate the earlier eluting peak fraction (~12.5 mL). Data were analyzed using Astra software (version 7.2.2; Wyatt Technology).

### Sacculi preparation

An established procedure was adopted (64), albeit with significant changes, to purify the PG of the strains under investigation. In summary, 200 mL cultures were grown to an OD_595_ of 0.5, and cells were harvested at 3,000 *× g* for 15 min. Pellets were boiled with magnetic stirring with 5% SDS for 3 h and then left overnight stirring at RT. Subsequently, the sacculi were pelleted by ultracentrifugation for 10 min at 20°C and 150,000* × g* and resuspended in 3 mL H_2_O. This was repeated three times to remove all SDS.

### *E. faecalis* peptidoglycan isolation

To isolate PG from *E. faecalis*, SDS-free pellets from sacculi preparation were resuspended in 1 mL Tris-HCl (100 mM, pH 7.5) and then glass beads measuring 0.1 mm in diameter were added (200 mg) and subjected to 10 min of vortexing at 4°C. The samples were subjected to a short spin (1 min) at 2,000 *× g* to allow precipitation of the glass beads and debris. Carefully, the supernatant was recovered and ultracentrifuged at 150,000* × g* for 10 min at 20°C. After centrifugation, the pellets were resuspended in 1 mL Tris-HCl, 100 mM, pH 7.5, and subjected to 2 h of treatment with 40 µL 1 M MgSO_4_, 2 µL RNase A (500 µg mL^−1^), and 1 µL DNase I (100 µg mL^−1^), followed by incubation with 50 µL CaCl_2_ and 100 µL trypsin (2 mg mL^−1^) for 16 h with magnetic stirring. Digestion was inactivated by adding 200 µL SDS 10% (wt/vol) and boiling for 10 min. Samples were centrifuged at 150,000 *× g* for 10 min at 20°C and washed with MilliQ water each time until the SDS was completely removed. SDS-free pellets were treated with 1 mL of 8 M LiCl for 10 min at 37°C and centrifuged again at 150,000* × g* for 10 min at 20°C. Pellets were resuspended with 1 mL EDTA 100 mM for 10 min at 37°C and centrifuged again at 150,000* × g* for 10 min at 20°C to remove EDTA. Pellets were resuspended with 1 mL of acetone for 10 min at RT and centrifuged again at 150,000* × g* for 10 min at 20°C. Teichoic acids were eliminated from the pellets by resuspension in 1 mL of 48% hydrofluoric acid and shaking incubation for 48 h at 4°C. After four rounds of centrifugation (150,000* × g* for 10 min at 20°C), each time washing the pellet with 3 mL cold MilliQ water, the pellets containing PG sacculi were obtained.

### *V. cholerae* peptidoglycan isolation

For PG isolation from *V. cholerae*, SDS-free pellets from sacculi preparation were resuspended in 100 mM Tris-HCl (pH 8), and proteinase K was added to a final concentration of 20 µg/mL and these were incubated for 1 h at 37°C. The reaction was stopped by adding 100 µL 10% SDS and boiling for 5 min. Sacculi were pelleted by ultracentrifugation for 10 min at 20°C and 150,000* × g* three times and each time washed with 3 mL of MiliQ water. Finally, the SDS-free PG sacculi were obtained.

### *In vitro* activity assays and sample preparation for liquid chromatography-mass spectrometry analysis

Protein samples were first purified on a Superdex 200 Increase 10/300 Gl column equilibrated with 300 mM NaCl, 50 mM Tris-HCl, pH 7.5. Pellets containing purified PG sacculi were resuspended in hydrolase buffer (100 mM NaPO_4_, 2 mM NaN_3_, 100 mM NaCl, pH 7.5) and Muramidase, Slt70_Ec_, PrgK_EC_, PrgK_LytM_, PrgK_SLT_, PrgK_CHAP_, PrgK_SLT-CHAP_ (including all the variants and combinations) were added to the reaction at a final concentration of 100 µg/mL. When TCEP was used, it was added at a final concentration of 10 mM. All the reactions were incubated at 37°C overnight (12–16 h) and subsequently stopped by boiling the reaction for 5 min. Once the samples were cooled, 20 µL of borate buffer (0.5 M boric acid adjusted to pH 9 with NaOH) was added (per 100 µL of reaction) to adjust the pH to alkaline. Next, 10 µL of 2 M NaBH_4_ (a mild reducing agent) was added and the sample was incubated for 30 min at 20°C. Finally, the pH of the sample was adjusted to 2–3 with 25% (vol/vol) orthophosphoric acid and the samples were filtered (0.2 µm pore size) for analysis.

### Peptidoglycan analysis

UPLC was used to perform chromatographic analyses of muropeptides. The UPLC system (Waters) was fitted with an analytical column (BEH C18 column; 130 Å, 1.7 µm, 2.1 mm by 150 mm; Waters, USA) and a trapping cartridge precolumn (SecurityGuard ULTRA Cartridge UHPLC C18 2.1 mm, Phenomenex). Muropeptides were identified using an ACQUITY UPLC UV-visible detector to measure absorbance at 204 nm. Muropeptides were separated over 15 min at a flow rate of 0.25 mL/min using a linear gradient from buffer A (water + 0.1% [vol/vol] formic acid) to buffer B (acetonitrile 100% [vol/vol ]+ 0.1% [vol/vol] formic acid). Muropeptides were quantified from their integrated areas using standards, which were samples at known concentrations. Using a Xevo G2-XS Q-tof system (Waters Corporation, USA), MS-MS/MS analysis was used to confirm the identity of the muropeptides. Positive ionization mode was used to operate the instrument. Muropeptide detection was carried out by mass spectrometry elevated energy (MS^E^) with the following parameters: capillary voltage at 3.0 kV, source temperature at 120°C, desolvation temperature at 350°C, sample cone voltage at 40 V, cone gas flow at 100 L/h, desolvation gas flow at 500 L/h, and collision energy (CE): low CE: 6 eV and high CE ramp: 15–40 eV. This allowed for the simultaneous acquisition of precursor and product ion data. A scan speed of 0.25 s/s was used to acquire mass spectra. The scan ranged from 100 to 2,000 *m*/*z*. Software programs like Masslynx or UNIFI were used for data processing and acquisition (Waters Corp.)

## Data Availability

Atomic coordinates and structure factors of the LytM domain have been deposited in the Protein Data Bank (PDB Code: 8S0U).

## SUPPLEMENTAL MATERIAL

Supplemental material mbio.00488-24-s0001.pdf Supplemental figures and tables.

ASM does not own the copyrights to Supplemental Material that may be linked to, or accessed through, an article. The authors have granted ASM a non-exclusive, world-wide license to publish the Supplemental Material files. Please contact the corresponding author directly for reuse.
