# Supplementary material for: Breaking barriers: pCF10 type 4 secretion system relies on a self-regulating muramidase to modulate the cell wall
Source: mBio. 2024 Jun 28;15(8):e00488-24. doi: 10.1128/mbio.00488-24 (PMC11323569; doi:10.1128/mbio.00488-24)
Supplement: Supplemental material — Supplemental figures and tables. [file mbio.00488-24-s0001.pdf]

**Supplementary information**

Breaking Barriers: pCF10 Type 4 Secretion System relies on a self-regulating muramidase to modulate the cell wall.

Wei-Sheng Sun[1,2], Gabriel Torrens[3], Josy ter Beek[1,2,#], Felipe Cava[3] and Ronnie P-A Berntsson[1,2,#]

[1] Department of Medical Biochemistry and Biophysics, Umeå University, SE-90187 Umeå, Sweden

[2] Wallenberg Centre for Molecular Medicine & Umeå Centre for Microbial Research, Umeå University, Umeå, Sweden

[3] Department of Molecular Biology and Laboratory for Molecular Infection Medicine Sweden, Umeå Centre for Microbial Research, SciLifeLab, Umeå University, Umeå, Sweden.

[#] Correspondence should be addressed to R.P-A.B. (email: ronnie.berntsson@umu.se) or J.t.B. (email: josy.beek@umu.se)

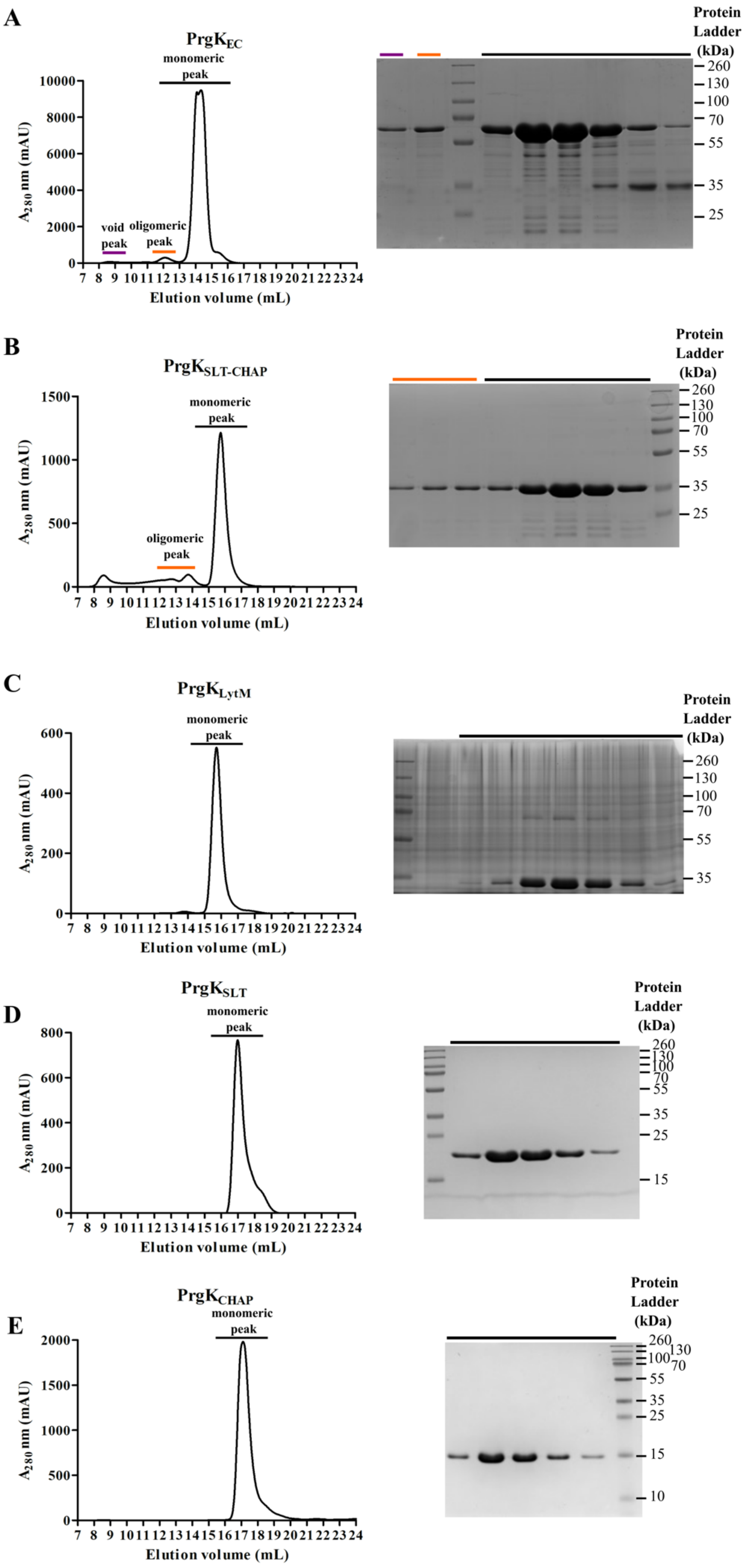
A
PrgK$_{EC}$
monomeric peak
void peak
oligomeric peak
$A_{280}$ nm (mAU)
Elution volume (mL)
Protein Ladder (kDa)
260
130
100
70
55
35
25
B
PrgK$_{SLT\text{-}CHAP}$
monomeric peak
oligomeric peak
$A_{280}$ nm (mAU)
Elution volume (mL)
Protein Ladder (kDa)
260
130
100
70
55
35
25
C
PrgK$_{LytM}$
monomeric peak
$A_{280}$ nm (mAU)
Elution volume (mL)
Protein Ladder (kDa)
260
130
100
70
55
35
D
PrgK$_{SLT}$
monomeric peak
$A_{280}$ nm (mAU)
Elution volume (mL)
Protein Ladder (kDa)
260
130
100
70
55
35
25
15
E
PrgK$_{CHAP}$
monomeric peak
$A_{280}$ nm (mAU)
Elution volume (mL)
Protein Ladder (kDa)
260
130
100
70
55
35
25
15
10

**Supplementary figure 1**. Representative elution profiles from size exclusion chromatography (left panels) and corresponding Coomassie-blue stained SDS-PAGE (right panels) of PrgK variants. Additional peaks other than the main peaks are marked with the same color in the left and right panels: A) $PrgK_{EC}$, consisting of residue 273-871, B) $PrgK_{SLT-CHAP}$, consisting of residue 530-871, C) $PrgK_{LytM}$, consisting of residue 273-529, D) $PrgK_{SLT}$, consisting of residue 539-723, E) $PrgK_{CHAP}$, consisting of residue 723-871.

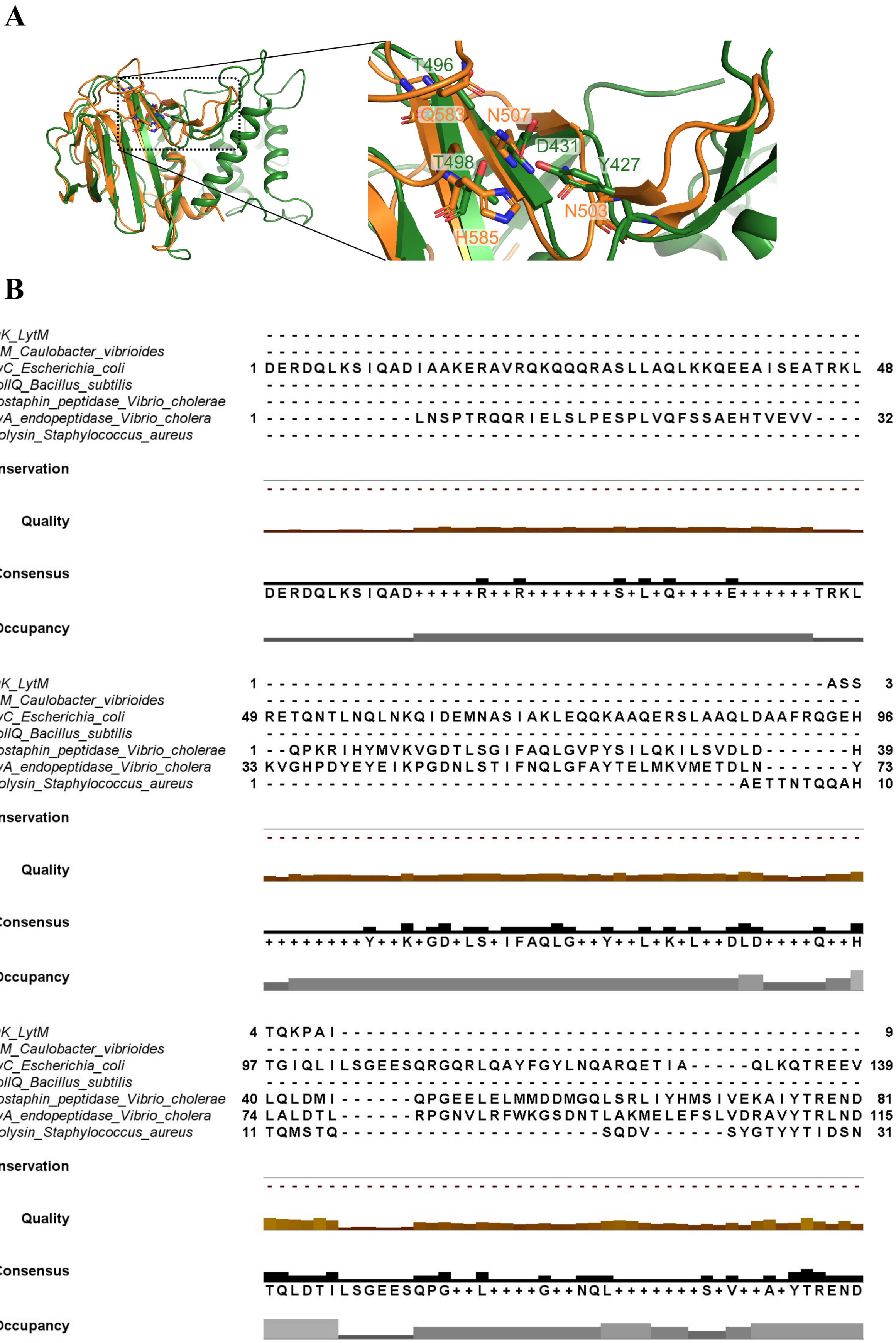
A
T496
Q583
N507
D431
Y427
T498
N503
H585
B
PrgK_LytM ------------------------------------------------
DipM_Caulobacter_vibrioides ------------------------------------------------
EnvC_Escherichia_coli 1 DERDQLKSIQADIAAKERAVRQKQQQRASLLAQLKKQEEAISEATRKL 48
SpoIIQ_Bacillus_subtilis ------------------------------------------------
lysostaphin_peptidase_Vibrio_cholerae ------------------------------------------------
ShyA_endopeptidase_Vibrio_cholera 1 ------------LNSPTRQQRIELSLPESPLVQFSSAEHTVEVV---- 32
autolysin_Staphylococcus_aureus ------------------------------------------------
Conservation
Quality
Consensus
DERDQLKSIQAD+++++R++R+++++++S+L+Q++++E++++++TRKL
Occupancy
PrgK_LytM 1 ---------------------------------------------ASS 3
DipM_Caulobacter_vibrioides ------------------------------------------------
EnvC_Escherichia_coli 49 RETQNTLNQLNKQIDEMNASIAKLEQQKAAQERSLAAQLDAAFRQGEH 96
SpoIIQ_Bacillus_subtilis ------------------------------------------------
lysostaphin_peptidase_Vibrio_cholerae 1 --QPKRIHYMVKVGDTLSGIFAQLGVPYSILQKILSVDLD-------H 39
ShyA_endopeptidase_Vibrio_cholera 33 KVGHPDYEYEIKPGDNLSTIFNQLGFAYTELMKVMETDLN-------Y 73
autolysin_Staphylococcus_aureus 1 --------------------------------------AETTNTQQAH 10
Conservation
Quality
Consensus
++++++++Y++K+GD+LS+IFAQLG++Y++L+K+L++DLD++++Q++H
Occupancy
PrgK_LytM 4 TQKPAI------------------------------------------ 9
DipM_Caulobacter_vibrioides ------------------------------------------------
EnvC_Escherichia_coli 97 TGIQLILSGEESQRGQRLQAYFGYLNQARQETIA-----QLKQTREEV 139
SpoIIQ_Bacillus_subtilis ------------------------------------------------
lysostaphin_peptidase_Vibrio_cholerae 40 LQLDMI------QPGEELELMMDDMGGQLSRLIYHMSIVEKAIYTREND 81
ShyA_endopeptidase_Vibrio_cholera 74 LALDTL------RPGNVLRFWKGSDNTLAKMELEFSLVDRAVYTRLND 115
autolysin_Staphylococcus_aureus 11 TQMSTQ--------------------SQDV------SYGTYYTIDSN 31
Conservation
Quality
Consensus
TQLDTILSGEESQPG++L++++G++NQL+++++++S+V++A+YTREND
Occupancy

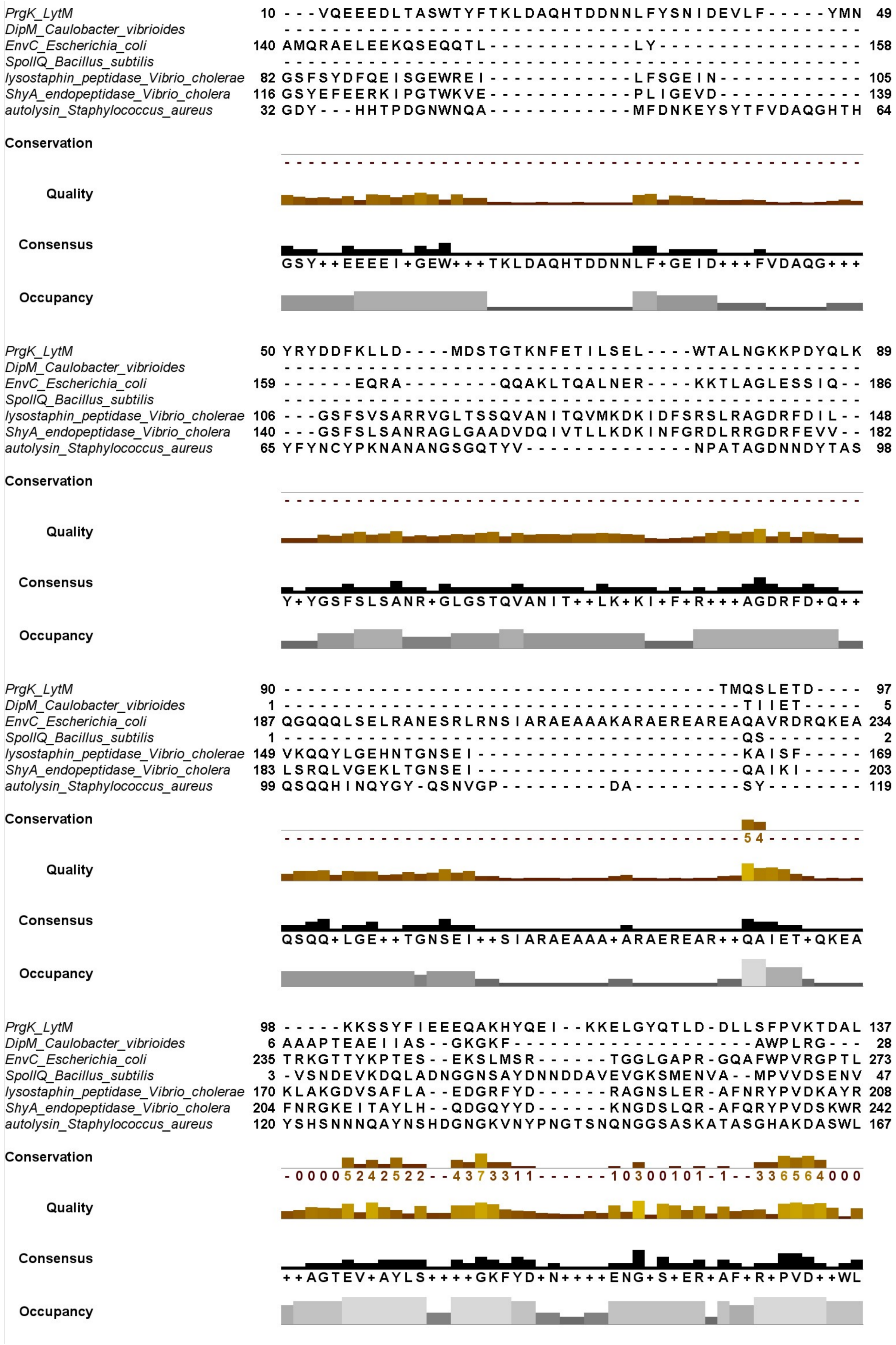
PrgK_LytM 10 - - - VQEEEDLTASWTYFTKLDAQHTDDNNLFYSNIDEVLF - - - - - YMN 49
DipM_Caulobacter_vibrioides
EnvC_Escherichia_coli 140 AMQRAELEEKQSEQQTL - - - - - - - - - - - - LY - - - - - - - - - - - - - - - - - 158
SpoIIQ_Bacillus_subtilis
lysostaphin_peptidase_Vibrio_cholerae 82 GSFSYDFQEISGEWREI - - - - - - - - - - - - LFSGEIN - - - - - - - - - - - - 105
ShyA_endopeptidase_Vibrio_cholera 116 GSYEFEERKIPGTWKVE - - - - - - - - - - - PLIGEVD - - - - - - - - - - - - 139
autolysin_Staphylococcus_aureus 32 GDY - - - HHTPDGNWNQA - - - - - - - - - - - - MFDNKEYSYTFVDAQGHTH 64
Conservation
Quality
Consensus
GSY++EEEEI+GEW+++TKLDAQHTDDNNLF+GEID+++FVDAQG+++
Occupancy
PrgK_LytM 50 YRYDDFKLLD - - - - MDSTGTKNFETILSEL - - - - WTALNGKKPDYQLK 89
DipM_Caulobacter_vibrioides
EnvC_Escherichia_coli 159 - - - - - - EQRA - - - - - - - - QQAKLTQALNER - - - - KKTLAGLESSIQ - - 186
SpoIIQ_Bacillus_subtilis
lysostaphin_peptidase_Vibrio_cholerae 106 - - - GSFSVSARRVGLTSSQVANITQVMKDKIDFSRSLRAGDRFDIL - - 148
ShyA_endopeptidase_Vibrio_cholera 140 - - - GSFSLSANRAGLGAADVDQIVTLLKDKINFGRDLRRGDRFEVV - - 182
autolysin_Staphylococcus_aureus 65 YFYNCYPKNANANGSGQTYYV - - - - - - - - - - - - - - NPATAGDNNDYTAS 98
Conservation
Quality
Consensus
Y+YGSFSLSANR+GLGSTQVANIT++LK+KI+F+R+++AGDRFD+Q++
Occupancy
PrgK_LytM 90 - - - - - - - - - - - - - - - - - - - - - - - - - - - - - - - - - - - TMQSLETD - - - - 97
DipM_Caulobacter_vibrioides 1 - - - - - - - - - - - - - - - - - - - - - - - - - - - - - - - - - - - - TIIET - - - - - - 5
EnvC_Escherichia_coli 187 QGQQQLSELRANESRLRNSIARAEAAAKARAEREAREAQAVRDRQKEA 234
SpoIIQ_Bacillus_subtilis 1 - - - - - - - - - - - - - - - - - - - - - - - - - - - - - - - - - - - - - QS - - - - - - - - 2
lysostaphin_peptidase_Vibrio_cholerae 149 VKQQYLGEHNTGNSEI - - - - - - - - - - - - - - - - - - - - - - - KAISF - - - - - 169
ShyA_endopeptidase_Vibrio_cholera 183 LSRQLVGEKLTGNSEI - - - - - - - - - - - - - - - - - - - - - - - QAIKI - - - - - 203
autolysin_Staphylococcus_aureus 99 QSQQHINQYGY - QSNVGP - - - - - - - - - DA - - - - - - - - - SY - - - - - - - - 119
Conservation
54
Quality
Consensus
QSQQ+LGE++TGNSEI++SIARAEAAA+ARAEREAR++QAIET+QKEA
Occupancy
PrgK_LytM 98 - - - - - KKSSYFIEEEQAKHYQEI - - KKELGYQTLD - DLLSFPVKTDAL 137
DipM_Caulobacter_vibrioides 6 AAAPTEAEIIAS - - GKGKF - - - - - - - - - - - - - - - - - - AWPLRG - - - 28
EnvC_Escherichia_coli 235 TRKGTTYKPTES - - EKSLMSR - - - - - TGGLGAPR - GQAFWPVRGPTL 273
SpoIIQ_Bacillus_subtilis 3 - VSNDEVKDQLADNGGNSAYDNNDDAVEVGKSMENVA - - MPVVDSENV 47
lysostaphin_peptidase_Vibrio_cholerae 170 KLAKGDVSAFLA - - EDGRFYD - - - - - - RAGNSLER - AFNRYPVDKAYR 208
ShyA_endopeptidase_Vibrio_cholera 204 FNRGKEITAYLH - - QDGQYYD - - - - - - KNGDSLQR - AFQRYPVDSKWR 242
autolysin_Staphylococcus_aureus 120 YSHSNNNQAYNSHDGNGKVNYPNGTSNQNGGSASKATASGHAKDASWL 167
Conservation
-00005242522--4373311------10300101-1--3365640000
Quality
Consensus
++AGTEV+AYLS++++GKFYD+N++++ENG+S+ER+AF+R+PVD++WL
Occupancy

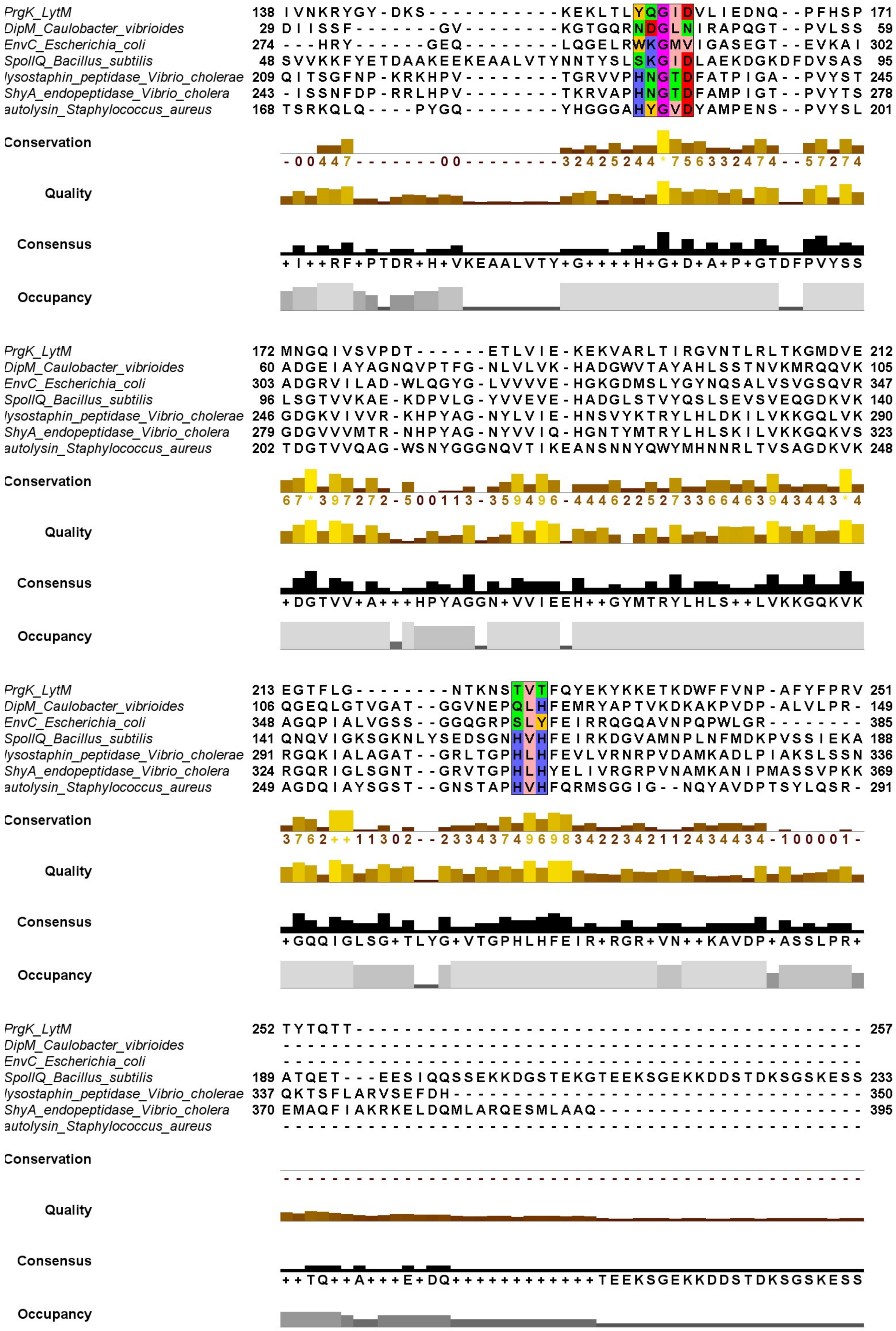
PrgK_LytM 138 IVNKRYGY-DKS-----------KEKLTLYQGIDVLIEDNQ--PFHSP 171
DipM_Caulobacter_vibrioides 29 DIISSF-------GV--------KGTGQRNDGLNIRAPQGT--PVLSS 59
EnvC_Escherichia_coli 274 ---HRY------GEQ--------LQGELRWKGMVIGASEGT--EVKAI 302
SpoIIQ_Bacillus_subtilis 48 SVVKKFYETDAAKEEKEAALVTYNNTYSLSKGIDLAEKDGKDFDVSAS 95
lysostaphin_peptidase_Vibrio_cholerae 209 QITSGFNP-KRKHPV--------TGRVVPHNGTDFATPIGA--PVYST 245
ShyA_endopeptidase_Vibrio_cholera 243 -ISSNFDP-RRLHPV--------TKRVAPHNGTDFAMPIGT--PVYTS 278
autolysin_Staphylococcus_aureus 168 TSRKQLQ----PYGQ--------YHGGGAHYGVDYAMPENS--PVYSL 201
Conservation
-00447--------00--------32425244*756332474--57274
Quality
Consensus
+I++RF+PTDR+H+VKEAALVTY+G++++H+G+D+A+P+GTDFPVYSS
Occupancy
PrgK_LytM 172 MNGQIVSVPDT------ETLVIE-KEKVARLTIRGVNTLRLTKGMDVE 212
DipM_Caulobacter_vibrioides 60 ADGEIAYAGNQVPTFG-NLVLVK-HADGWVTAYAHLSSTNVKMRQQVK 105
EnvC_Escherichia_coli 303 ADGRVILAD-WLQGYG-LVVVVE-HGKGDMSLYGYNQSALVSVGSQVR 347
SpoIIQ_Bacillus_subtilis 96 LSGTVVKAE-KDPVLG-YVVEVE-HADGLSTVYQSLSEVSVEQGDKVK 140
lysostaphin_peptidase_Vibrio_cholerae 246 GDGKVIVVR-KHPYAG-NYLVIE-HNSVYKTRYLHLDKILVKKGQLVK 290
ShyA_endopeptidase_Vibrio_cholera 279 GDGVVVMTR-NHPYAG-NYVVIQ-HGNTYMTRYLHLSKILVKKGQKVS 323
autolysin_Staphylococcus_aureus 202 TDGTVVQAG-WSNYGGGNQVTIKEANSNNYQWYMHNNRLTVSAGDKVK 248
Conservation
67*397272-500113-359496-4446225273366463943443*4
Quality
Consensus
+DGTVV+A+++HPYAGGN+VVIEEH++GYMTRYLHLS++LVKKGQKVK
Occupancy
PrgK_LytM 213 EGTFLG--------NTKNSTVTFQYEKYKKETKDWFFVNP-AFYFPRV 251
DipM_Caulobacter_vibrioides 106 QGEQLGTVGAT--GGVNEPQLHFEMRYAPTVKDKAKPVDP-ALVLPR- 149
EnvC_Escherichia_coli 348 AGQPIALVGSS--GGQGRPSLYFEIRRQGQAVNPQPWLGR-------- 385
SpoIIQ_Bacillus_subtilis 141 QNQVIGKSGKNLYSEDSGNHVHFEIRKDGVAMNPLNFMDKPVSSIEKA 188
lysostaphin_peptidase_Vibrio_cholerae 291 RGQKIALAGAT--GRLTGPHLHFEVLVRNRPVDAMKADLPIAKSLSSN 336
ShyA_endopeptidase_Vibrio_cholera 324 RGQRIGLSGNT--GRVTGPHLHYELIVRGRPVNAMKANIPMASSVPKK 369
autolysin_Staphylococcus_aureus 249 AGDQIAYSGST--GNSTAPHVHFQRMSGGIG--NQYAVDPTSYLQSR- 291
Conservation
3762++11302--233437496983422342112434434-100001-
Quality
Consensus
+GQQIGLSG+TLYG+VTGPHLHFEIR+RGR+VN++KAVDP+ASSLPR+
Occupancy
PrgK_LytM 252 TYTQTT------------------------------------------ 257
DipM_Caulobacter_vibrioides ------------------------------------------------
EnvC_Escherichia_coli ------------------------------------------------
SpoIIQ_Bacillus_subtilis 189 ATQET---EESIQQSSEKKDGSTEKGTEEKSGEKKDDSTDKSGSKESS 233
lysostaphin_peptidase_Vibrio_cholerae 337 QKTSFLARVSEFDH---------------------------------- 350
ShyA_endopeptidase_Vibrio_cholera 370 EMAQFIAKRKELDQMLARQESMLAAQ---------------------- 395
autolysin_Staphylococcus_aureus ------------------------------------------------
Conservation
------------------------------------------------
Quality
Consensus
++TQ++A+++E+DQ++++++++++++TEEKSGEKKDDSTDKSGSKESS
Occupancy

**Supplementary figure 2**. Comparison of $PrgK_{LytM}$ to homologs. A) Left panel: Structure superimposition of *C. vibrioides* DipM (PDB Code: 7QRL, residues 461-609) (orange) to $PrgK_{LytM}$ (green). The degenerate catalytic centers of DipM and $PrgK_{LytM}$ are highlighted in the dashed box and enlarged in the right panel, in which the residues corresponding to the HXXXD and HXH motifs are displayed as sticks. B) Primary sequence alignment of $PrgK_{LytM}$ homologues. The HXXXD and HXH motifs that are essential for catalysis are highlighted in colored squares. Homologous proteins include catalytically degenerate ones: *C. vibrioides* DipM (7qrl, 461-609), *E. coli* EnvC (6tpi, 35-419), and *B. subtilis* SpIIQ (3tuf, 43-283); catalytically active ones are exemplified by lysostaphin peptidase from *Vibrio cholerae* (2gu1, 63-412), *V. cholerae* ShyA endopeptidase (6u2a, 36-430), and *S. aureus* autolysin LytM (1qwy, 26-316). The consensus sequence is displayed as logos below the individual sequences. Numbers on the left and right are scales, not corresponding to the actual residue numbers.

**A**

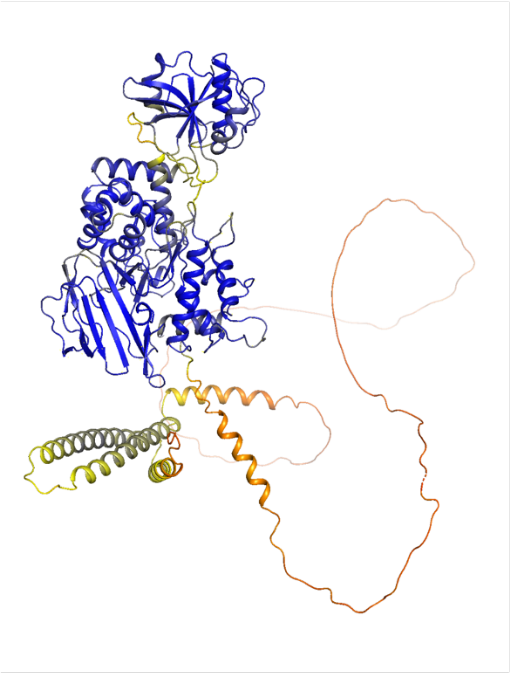

**B**

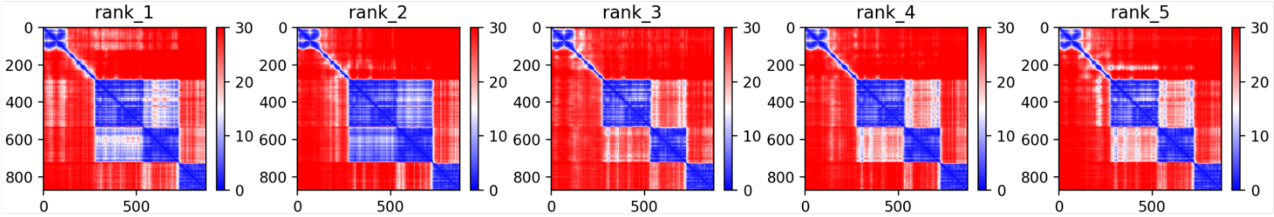


**Supplementary figure 3**. AlphaFold2 model of full length PrgK. A) pLDDT colored full length PrgK, including the intracellular domain. Colors range from red (low quality) to blue (high quality). B) PAE plots of the top five models generated by AlphaFold2. The model is reasonably confident in the placement of the LytM and SLT domain in relation to each other, but not the remaining domains.

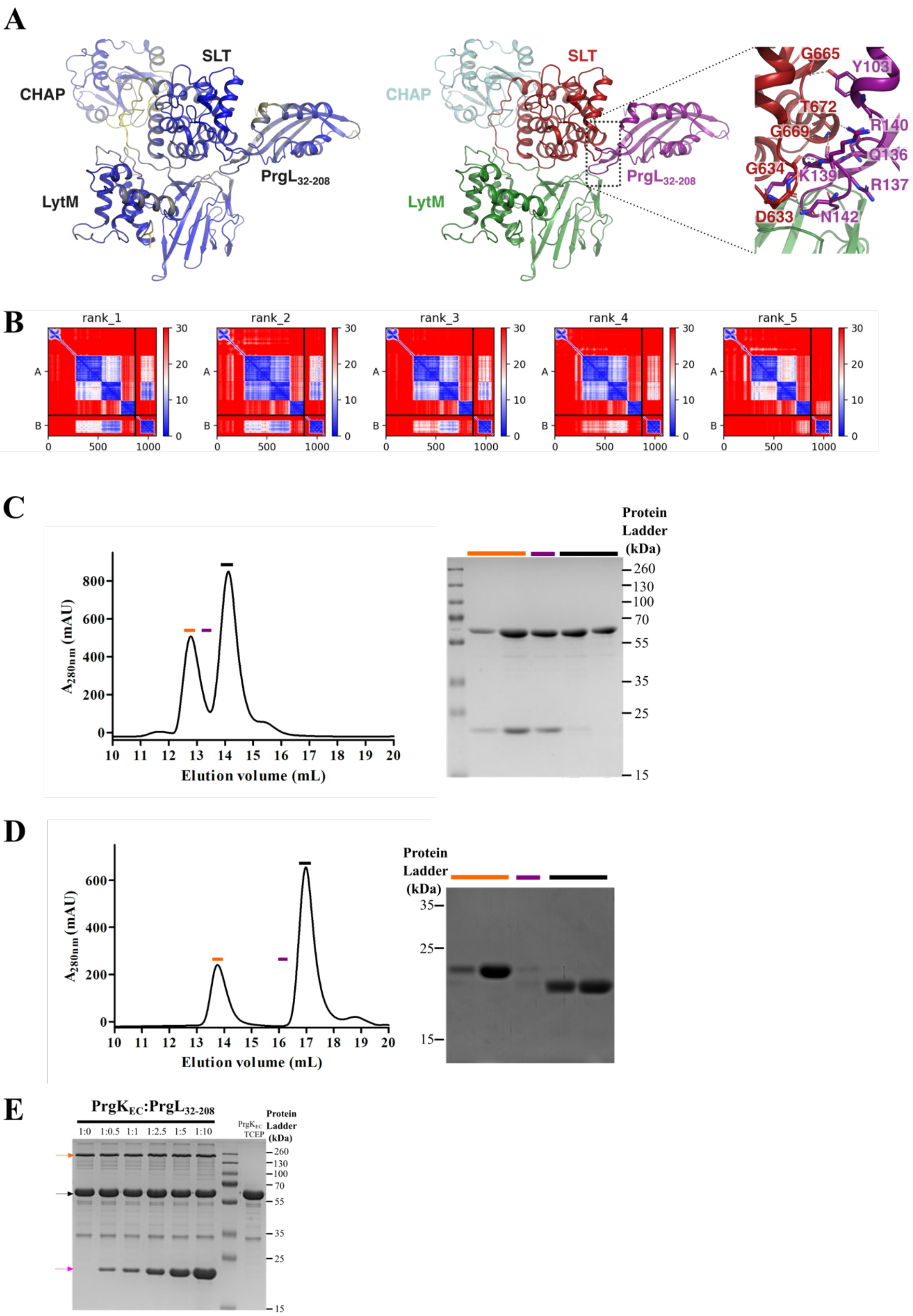


**Supplementary figure 4**. PrgK-PrgL protein-protein interaction. A) AlphaFold2 multimer prediction of protein complex formed between PrgK and PrgL. Left: colored by pLDDT, right: colored by domains. B) PAE plots of the top five AlphaFold2 models of PrgK:PrgL. C) Left:

representative size exclusion chromatography elution profile (Superdex 200 Increase 10/300 GL column) of $PrgK_{EC}$ and $PrgL_{32-208}$ mixture. Right: SDS-PAGE of fractions covering the first peak (orange), second peak (black), and the interval (purple). D) Left: representative size exclusion chromatography elution profile (Superdex 200 Increase 10/300 GL column) of $PrgK_{MUR(SLT)}$ and $PrgL_{32-208}$ mixture. Right: SDS-PAGE of fractions covering the first peak (orange), second peak (black), and the interval (purple). **E**) *In vitro* dimerization of $PrgK_{EC}$ is not altered in the presence of $PrgL_{32-208}$. The protein bands of dimeric $PrgK_{EC}$ (indicated by orange arrow) and monomeric $PrgK_{EC}$ (indicated by black arrow) in the presence of increasing amounts of $PrgL_{32-208}$ (indicated by purple arrows). The molar ratios of $PrgK_{EC}$:$PrgL_{32-208}$ are indicated above each lane. $PrgK_{EC}$ incubated with TCEP, which reduces the dimer to monomers, is shown as a control (final lane).

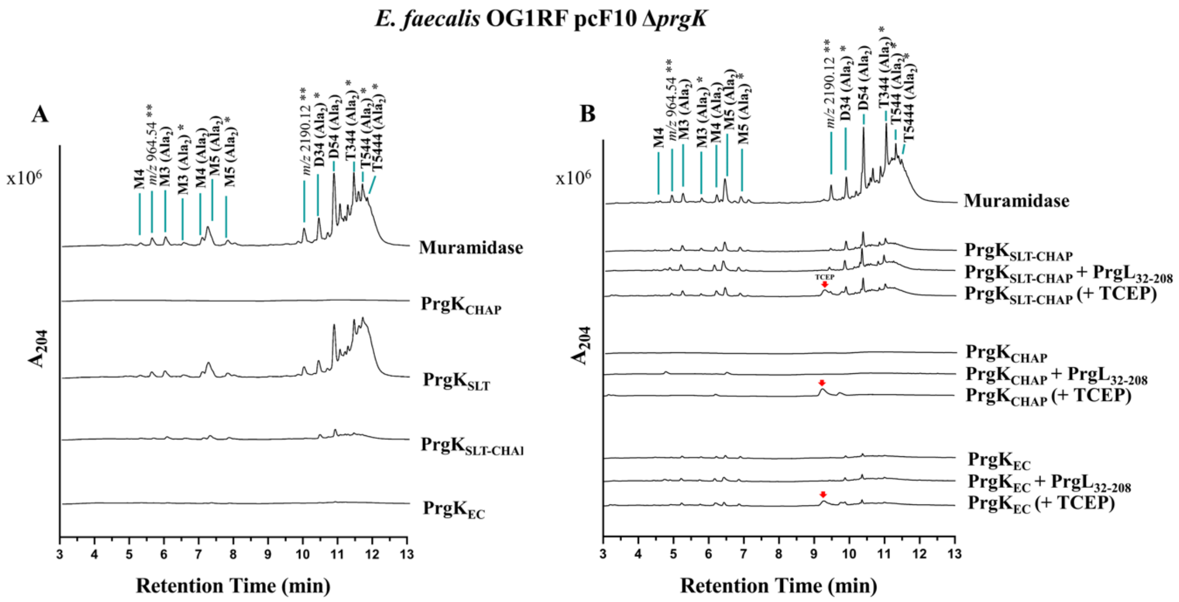


**Supplementary figure 5**. Comparing cell wall muropeptides generated by PrgK domains. Chromatograms of muropeptides released following *E. faecalis* OG1RF:pCF10Δ*prgK* PG treatment with the indicated PrgK enzymes variants (A) and with addition of $PrgL_{32-208}$ or TCEP (B). Identified muropeptides are indicated above their corresponding peak (See table 2 for further information). Unknown muropeptides are marked for their corresponding *m/z* values. Red arrows indicate the peak for TCEP.

* Gln/Glu. It is arbitrarily to assign the amide and hydroxyl roles to either peptide stem.

** Precise structure unknown

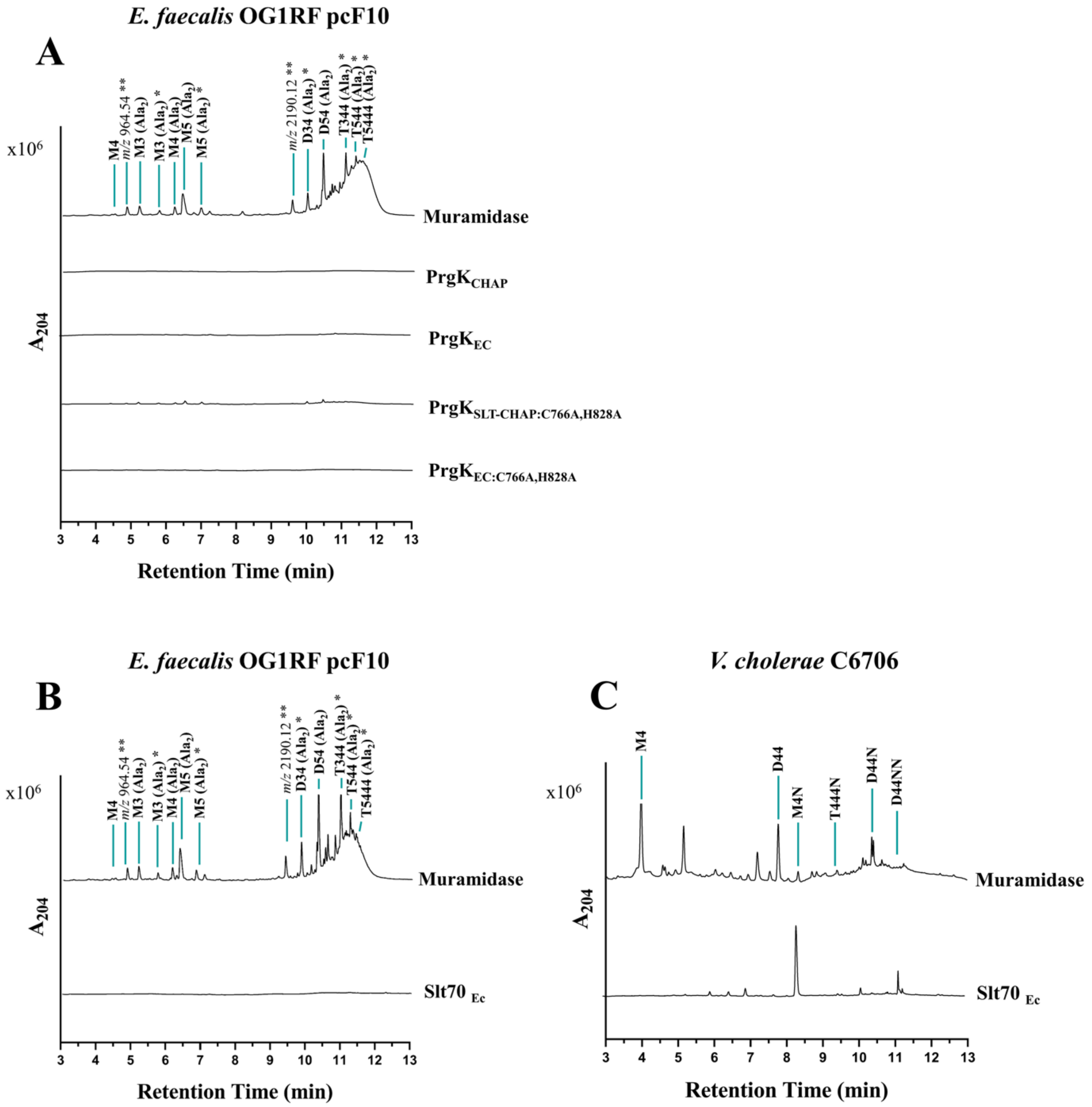


**Supplementary figure 6.** Activity of $PrgK_{SLT}$. A) Mutating the active site residues C766 and H828 to alanines in the CHAP domain does not change the activity of PrgK B) Slt70 from *E. coli* does not function on sacculi from *E. faecalis*. C) Slt70 from *E. coli* does cleave sacculi isolated from *V. cholerae*.

* Gln/Glu. It is arbitrarily to assign the amide and hydroxyl roles to either peptide stem.

** Precise structure unknown

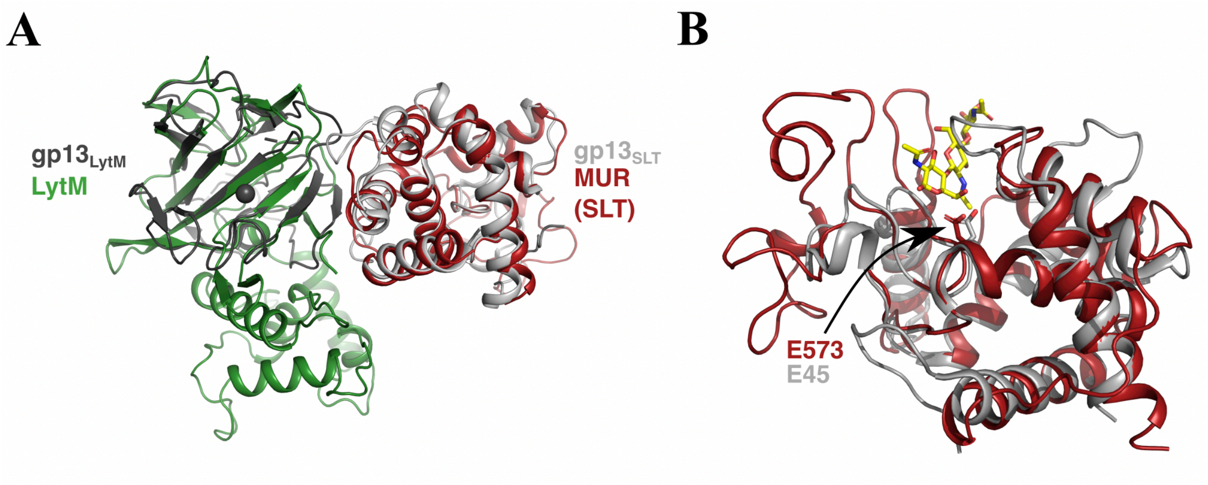


**Supplementary Figure 7.** Comparison of $PrgK_{LytM+MUR}$ AlphaFold2 model with the crystallized cell-wall degrading enzyme gp13 of bacteriophage Φ29 (PDB code:3CSQ). A) The SLT domain of gp13 (light gray) and the LytM domain of gp13 (dark gray) superimpose well onto $PrgK_{MUR}$ and $PrgK_{LytM}$ (RMSD of 1.5 Å and 2.4 Å), respectively (the gp13 domains were superimposed individually onto PrgK, and do not represent the correct orientation in the gp13 crystal structure). In contrast to $PrgK_{LytM}$, the $gp13_{LytM}$ domain has a functional active site, and has a $Zn^{2+}$ bound. (shown as sphere) B) Here only visualizing the SLT domain of gp13 superimposed on the MUR domain of PrgK. The sugar product in gp13 is shown in yellow sticks, and the active site Glutamic acid, which is conserved in the MUR domain, is highlighted. The loops lining the active site deviate greatly between the proteins.

Table S1. Table of bacterial strains, plasmids and nucleotides used.

| Strain, plasmid, or oligonucleotide | Relevant feature(s) or sequence | Source/ reference |
|---|---|---|
| **Strains** | | |
| *E. coli* | | |
| BL21 DE3 | Laboratory strain for expressing gene driven by T7 promoter | New England Biolabs |
| Top10 | laboratory strain for cloning | Thermofisher Scientific |
| *E. faecalis* | | |
| OG1RF | parental strain, $Rif^R$, $Fus^R$ | Dunny et al., 1981 |
| **Plasmids** | | |
| pCF10 | cCF10-inducible conjugative plasmid, $Tet^R$ | Dunny et al., 1981 |
| pCF10Δ*prgK* | pCF10 plasmid with *prgL* deletion, $Tet^R$ | Gomez et al., 2014 |
| pINIT_kan | FX-cloning intermediate vector, $Kan^R$ | Geertsma et al., 2011 |
| p7XC3H | FX cloning *E. coli* expression vector with T7 promoter, fusing 10x His tags, $Kan^R$, $Cm^R$ | Geertsma et al., 2011 |
| p7XC3GH | FX cloning *E. coli* expression vector with T7 promoter, fusing 10x His tags and sfGFP in C-terminal, $Kan^R$, $Cm^R$ | Geertsma et al., 2011 |
| p7XNH3 | FX cloning *E. coli* expression vector with T7 promoter, fusing 10x His tags in N-terminal, $Kan^R$, $Cm^R$ | Geertsma et al., 2011 |
| p7XNH3-prgK$_{EC}$ | p7XNH3 expressing PrgK$_{EC,}$ residues 273-871 | This study |
| p7XNH3-prgK$_{EC:C766A}$ | p7XNH3 expressing PrgK$_{EC}$, mutant of C766A, residues 273-871 | This study |
| p7XNH3-prgK$_{EC:}$ C766A H828A | p7XNH3 expressing PrgK$_{EC,}$ mutant of C766A H828A, residues 273-871 | This study |
| p7XC3GH-prgK$_{SLT-}$ CHAP | p7XC3GH expressing PrgK$_{SLT-CHAP}$, residues 530-871 | This study |
| p7XC3GH-prgK$_{SLT-}$ CHAP: C766A H828A | p7XC3GH expressing PrgK$_{SLT-CHAP}$, mutant of C766A H828A, residues 530-871 | This study |
| p7XNH3-prgK$_{LytM}$ | p7XNH3 expressing PrgK$_{LytM}$, residues 273-529 | This study |
| p7XC3GH-prgK$_{SLT}$ | p7XC3GH expressing PrgK$_{SLT}$, residues 539-723 | This study |
| p7XC3GH-prgK$_{CHAP}$ | p7XC3GH expressing PrgK$_{CHAP}$, residues 723-871 | This study |
| p7XC3H_prgL$_{32-208}$ | p7XC3H expressing PrgL soluble part, residues 32-208 | Jäger et al., 2022 |
| pMSP3545S | *E. coli* and Gram-positive shuttle vector for nisin-inducible expression, $ery^R$, $spc^R$ | Chandler et al., 2005 |
| pMSP3545_prgK | pMSP3545 harboring full-length *prgK* gene | Gomez et al., 2014 |
| pMSP3545_prgK: C766A H828A | pMSP3545S harboring full-length *prgK* gene, with mutation of C766A H828A | Gomez et al., 2014 |
| pET28b::slt70 | pET28 expressing Slt70 | Espaillat *et al,* 2016 |
| BL21 pET28b::XNR_0208 | pET28 expressing Muramidase | Alvarez *et al.* 2016 |
| **Primers** | | |
| PrgK$_{EC}$-fw | 5’-ATATATGCTCTTCTAGTGCAAGTAGTACACAAAAACCAGCGATT -3’ | This study |
| PrgK$_{EC}$-rev | 5’-TATATAGCTCTTCCTGCATTTAGTCGCATAAAGTATTCGCC -3’ | This study |
| PrgK$_{LytM}$-rev | 5’-TATATAGCTCTTCATGCAGTCGTTTGAGTGTAGGTTACACGTGG -3’ | This study |
| PrgK$_{SLT-CHAP}$-fw | 5’-ATATATGCTCTTCTAGTCTTTTAGGAAGTGCAGATTTTTCTCCT -3’ | This study |

| | | |
|---|---|---|
| $PrgK_{SLT}$-fw | 5'-CAGTCAGCTCTTCTAGTGGTGCAAGTGTCGAAAAGAGAGCGCAA -3' | This study |
| $PrgK_{SLT}$-rew | 5'-TATATAGCTCTTCATGCACCCGATCGAGAAAAGAATGTAAACCA -3' | This study |
| $PrgK_{CHAP}$-fw | 5'-ATATATGCTCTTCTAGTGGTACGCCAATCGGTGGTTCTGGGAGT -3' | This study |
| $PrgK_{CHAP}$-rew | 5'-TATATAGCTCTTCCTGCATTTAGTCGCATAAAGTATTCGCC -3' | This study |
| PrgK_C766A_inv-F | 5'- GAATGCCACGTGGTATGTGTTTAATCGTTTTGCACAG -3' | This study |
| PrgK_C766A_inv-R | 5'- ACCACGTGGCATTCCCTGGCGCATACGCATTG -3' | This study |

**Table S2.** Molecular mass and proposed structure of muropeptides from *E. faecalis*

| Identity | Proposed Structure | Observed *m/z* | Observations |
|---|---|---|---|
| M4 | GlcNAc-MurNAc-L-Ala-D-iGln-L-Lys-D-Ala | 897.44 | [M+H]+ |
| ND | Precise structure unknown | 964.54 | [M+H]+ |
| M3 ($Ala_2$) | GlcNAc-MurNAc-L-Ala-D-iGln-L-Lys(-L-Ala-L-Ala) | 968.48 | [M+H]+ |
| M3 ($Ala_2$)* | GlcNAc-MurNAc-L-Ala-D-iGlu-L-Lys(-L-Ala-L-Ala) | 969.46 | [M+H]+ |
| M4 ($Ala_2$) | GlcNAc-MurNAc-L-Ala-D-iGln-L-Lys(-L-Ala-L-Ala)-D-Ala | 1039.52 | [M+H]+ |
| M5 ($Ala_2$) | GlcNAc-MurNAc-L-Ala-D-iGln-L-Lys(-L-Ala-L-Ala)-D-Ala-D-Ala | 1110.55 | [M+H]+ |
| M5 ($Ala_2$)* | GlcNAc-MurNAc-L-Ala-D-iGlu-L-Lys(-L-Ala-L-Ala)-D-Ala-D-Ala | 1111.53 | [M+H]+ |
| ND | Precise structure unknown | 2190.12 | [M+H]+ |
| D34 ($Ala_2$)* | M3 ($Ala_2$) - M4 ($Ala_2$) | 996.99 | [M+2H]2+ |
| D54 ($Ala_2$) | M5 ($Ala_2$) - M4 ($Ala_2$) | 1067.03 | [M+2H]2+ |
| T344 ($Ala_2$)* | M3 ($Ala_2$) - M4 ($Ala_2$) - M4 ($Ala_2$) | 1005.17 | [M+3H]3+ |
| T544 ($Ala_2$)* | M5 ($Ala_2$) - M4 ($Ala_2$) - M4 ($Ala_2$) | 1052.85 | [M+3H]3+ |
| T5444 ($Ala_2$)* | M5 ($Ala_2$) - M4 ($Ala_2$) - M4 ($Ala_2$) - M4 ($Ala_2$) | 1045.27 | [M+4H]4+ |

GlcNAc-MurNAc: N-acetylglucosamine and N-acetylmuramic acid; Ala, Alanine; Gln, Glutamine; Glu, Glutamic acid; Lys, Lysine.

* It is arbitrary to assign the amide and hydroxyl functions to either peptide stem.
